# Biomarker Levels of Toxic Metals among Asian Populations in the United States: NHANES 2011–2012

**DOI:** 10.1289/EHP27

**Published:** 2016-08-12

**Authors:** Hiroshi Awata, Stephen Linder, Laura E. Mitchell, George L. Delclos

**Affiliations:** 1 University of Texas Health Science Center at Houston School of Public Health, Houston, Texas, USA; 2 CH2M HILL, Inc., Houston, Texas, USA

## Abstract

**Introduction:**

The Centers for Disease Control and Prevention (CDC) recently found that Asians have considerably higher biomarker levels of cadmium, lead, mercury, and arsenic than whites, blacks, Mexican Americans, and other Hispanics in the United States.

**Objective:**

Our goal was to further evaluate the higher metal biomarker levels among Asians.

**Methods:**

Biomarker data (blood cadmium, blood lead, blood mercury, urinary total arsenic, and urinary dimethylarsinic acic) from individuals ≥ 6 years of age were obtained from the 2011–2012 National Health and Nutrition Examination Survey (NHANES). We compared geometric mean levels of these five metal biomarkers in Asians with those of four other NHANES race/ethnic groups (white, black, Mexican American, and other Hispanic), and across three Asian subgroups (Chinese, Asian Indian, and other Asian). We also evaluated associations between biomarker levels and sociodemographic, physical, dietary, and behavioral covariates across the Asian subgroups.

**Results:**

Asians had significantly higher levels of all five metal biomarkers than other race/ethnic groups (*p* < 0.05), regardless of sociodemographic, physical, dietary, behavioral, or geographic characteristics. We also found variations in biomarker levels across the Asian subgroups. In general, Asian Indians had lower levels than the other two Asian subgroups, except for blood lead. The following characteristics were found to be significant predictors of several biomarker levels: sex, age, education, birthplace, smoking, and fish consumption.

**Conclusions:**

Overall, the Asian group had the highest geometric mean biomarker levels for all of the five metal variables. Furthermore, we provided evidence that significant variations in the biomarker levels are present across the Asian subgroups in the United States.

**Citation:**

Awata H, Linder S, Mitchell LE, Delclos GL. 2017. Biomarker levels of toxic metals among Asian populations in the United States: NHANES 2011–2012. Environ Health Perspect 125:306–313; http://dx.doi.org/10.1289/EHP27

## Introduction

Cadmium, lead, mercury, and arsenic are among the most toxic environmental contaminants. The International Agency for Research on Cancer (IARC) classifies arsenic and cadmium as human carcinogens (Group 1), and lead and mercury (methylmercury) as possibly carcinogenic to humans (Group 2B) (IARC 2013). Although levels of exposure to these metals/metalloids (hereafter, collectively referred to simply as “metals”) have been generally decreasing in the United States, various adverse health effects, such as cardiovascular and developmental effects, damage to the nervous system, and kidney failure, have been associated with exposure to these metals at the current, relatively low, environmental exposure levels (Ferraro et al. 2010; Lebel et al. 1996; McLaine et al. 2013; Moon et al. 2013). The health effects of low-level exposures are also important because some of the effects have been regarded to have no safe exposure threshold (Anderson 1983; Jakubowski 2011). Therefore, exposure to these toxic metals still poses a significant public health risk, and it is vital to reduce overall exposure and subsequently health risks, especially for those highly exposed subpopulation groups.

Asian populations have considerably higher blood and urinary levels of these metals than other racial/ethnic groups (i.e., whites, blacks, and Hispanics) in the United States (CDC 2014; McKelvey et al. 2007). For example, based on a recent analysis of biomarker data by the Centers for Disease Control and Prevention (CDC) in 2014, the geometric mean blood mercury levels among Asians (1.86 μg/L) is four times greater than that of Mexican Americans (0.48 μg/L) (CDC 2014). Asian populations in the United States include multiple ethnic subgroups that are culturally, religiously, historically, and geographically diverse. Hence, the differences in these characteristics across subgroups may affect biomarker levels of these metals. However, this was not examined in the original CDC analysis.

The National Health and Nutrition and Examination Survey (NHANES) is a national population-based survey program conducted by the National Center for Health Statistics (NCHS) that assesses the health and nutritional status of the civilian noninstitutionalized general U.S. population. The NCHS collects data continuously and releases data every 2 years in a 2-year data cycle. An important addition to the most recent data cycle (i.e., NHANES 2011–2012) was that Asian populations were oversampled, and data for Asians were reported in a separate race category as opposed to being included in the “other” race category (NCHS 2013). Because studies evaluating the health and nutrition status among Asians on a national level are relatively scarce, the addition of the Asian category should allow researchers to investigate the health and nutrition status of this race group. Further, evaluation of exposure characteristics across Asian subgroups could help identify highly exposed subpopulations and also their potential exposure sources.

The objective of the present study was to expand the CDC’s analysis of biomarker data and further evaluate the higher metal biomarker levels among Asians by comparing the biomarker levels of four metals (cadmium, lead, mercury, and arsenic) in Asians with those of other racial and ethnic groups in the United States. We examined variations in biomarker levels of metals in the major Asian subgroups (Chinese and Asian Indian) in the United States and the association of biomarker levels with various demographic, socioeconomic, physical, dietary, behavioral, and geographical characteristics within the subgroups.

## Methods

### Data Source

NHANES data available through the CDC were used as the data source. NHANES recruits approximately 5,000 participants annually, using a complex, multistage, probability sampling design. The multistage sampling procedure includes sampling from four stages of geographical units (county, city block, household, and individual), where subsequent sampling occurs within the unit selected in the prior stage. Multiple samples can be drawn from the same unit (e.g., multiple individuals from one household). Self-reported demographic, socioeconomic, dietary, and health-related information is collected through interview and questionnaire, whereas medical examination and collection of biological specimens (blood/urine) for laboratory tests are administered by health professional and qualified staff at the mobile examination center. The NHANES data collection procedures are described in detail elsewhere (Johnson et al. 2014).

The majority of the NHANES data are publicly available and were obtained directly from the CDC web site (CDC 2015). Access to certain data sets is restricted to protect study participant confidentiality. The restricted data used in this study (i.e., Asian ancestry and geographical information of the participants) were accessed and analyzed at the CDC Research Data Center (RDC), following a strict NCHS protocol (NCHS 2012a). Data collection for NHANES was approved by the NCHS Research Ethics Review Board (ERB). Analysis of de-identified data from the survey is exempt from federal regulations for the protection of human research participants. Analysis of restricted data through the NCHS RDC was also approved by the NCHS ERB.

### Study Population

For this study, the study population was the general U.S. population (≥ 6 years of age), including both males and females and all racial and ethnic groups, except those categorized as “other” (i.e., Pacific Islanders, Native Americans/Alaskan Natives, and multiracial individuals). The “other” race group was excluded because of its small sample size and the heterogeneous nature of the group. Additionally, the non-Hispanic Asian group [Far East Asia, Southeast Asia, or South Asia/the Indian subcontinent (NCHS 2013)] was subdivided into Chinese (Chinese and Taiwanese), Asian Indian (Asian Indian, Bengalese, Bharat, Dravidian, East Indian, and Goanese), and Other Asians based on the answer to DMQ.336 in the NHANES’s survey questionnaire. When a participant selected multiple Asian ancestries (e.g., Chinese and Filipino), they were categorized into the “Other Asian” subgroup. Chinese and Asian Indians were selected because they are the two largest Asian subgroups. Each subgroup accounts for approximately 20% of the Asian population (Hoeffel et al. 2012). There was no oversampling of the specific subgroups within the Asian population performed in NHANES 2011–2012.

### Biomarker Data

We evaluated five biomarkers: blood cadmium (B-Cd), blood lead (B-Pb), blood mercury (B-Hg), urinary total arsenic (U-tAs) and urinary dimethylarsinic acid (U-DMA). Study participants age ≥ 1 year were eligible for collection of blood samples, whereas urinary samples were obtained from a randomly selected one-third subset of the participants (≥ 6 years old). Arsenic acid, arsenous acid, monomethylarsonic acid (MMA), and DMA are metabolites of inorganic arsenic. Although methylated species such as MMA and DMA can be metabolites of less harmful organic arsenic, these five inorganic arsenic metabolites are often summed to represent inorganic arsenic exposure. Because inorganic arsenic metabolites other than DMA typically have low frequency of detection (< 40%), we only evaluated biomarker levels of U-DMA in our study. Similar to the CDC study of metal biomarkers (CDC 2014), urinary metal concentrations were adjusted using the concentration of creatinine in urine to account for the effect of urinary dilution:

Creatinine-corrected urinary concentration (μg/g) = [100(*L* • *mg/dL* • *g*) × *metal concentration in urine* (*μg/L*)] ÷ [*creatinine in urine* (*mg/dL*)]

For samples with biomarker levels below the limit of detection (LOD), NHANES uses “fill values” (LOD divided by the square root of 2). In accordance with the *Fourth National Report on Human Exposure to Environmental Chemicals* (CDC 2014), we used these fill values in our analyses. The LOD for biomarker parameters used to establish the fill values were as follows: B-Cd, 0.16 μg/L; B-Pb, 0.25 μg/dL; B-Hg, 0.16 μg/L; U-tAs, 1.25 μg/L; U-DMA, 1.80 μg/L. The detection frequency of B-Cd ranged from 63% among Mexican Americans to 87% among Asians; for U-DMA, the detection frequency ranged from 73% among whites to 91% among Asians. The biomarker levels of three other metal variables presented a relatively high frequency of detection in all groups: B-Pb (≥ 98%), B-Hg (≥ 91%), U-tAs (≥ 91%). The biomarker data were log-transformed to reduce skewness. Detailed information about laboratory procedures including sample collection, storing, and handling of specimens, quality control, and instrument and equipment used for the chemical analyses can be found elsewhere (NCHS 2011a, 2011b, 2012b).

### Covariates

The covariates included in the analyses were sex, age, education, household income, birthplace, poverty–income ratio (PIR) according to the Department of Health and Human Services poverty guidelines (dichotomized based on the median value of 1.63) (DHHS 2013), body mass index (BMI) (underweight, < 18.5 kg/m^2^; normal weight, 18.5–< 25 kg/m^2^; overweight, 25–< 30 kg/m^2^; obese, ≥ 30 kg/m^2^), smoking (based on the tertile of serum cotinine level), fish consumption, urbanization classification based on 2013 NCHS urban–rural classification scheme for counties (Ingram and Franco 2014), and U.S. Census region. BMI was included based on the association between lower BMI and high B-Hg levels observed in previous studies (Buchanan et al. 2015; Rothenberg et al. 2015). For participants < 20 years of age, education level of the household reference person (frequently, the adult owner/renter of the residence) was used. BMI category was determined based on the CDC’s sex-specific 2000 BMI for-age growth charts for the age group < 20 years (underweight, < 5th percentile; normal weight, 5th–< 85th percentile; overweight, 85th–< 95th percentile; obese, ≥ 95th percentile). Table 1 provides details on the breakdown and response categories of each of these covariates.

**Table 1:** Characteristics of study participants [*n *(%) or %] with weighted percentage, NHANES 2011–2012.

Covariates^*a*^	Non-Hispanic white [2,374 (66.4)]	Non-Hispanic black [1,957 (12.2)]	Mexican American [920 (9.4)]	Other Hispanic [755 (6.9)]	Non-Hispanic Asian [945 (5.0)]	Asian subgroups (%)^*b*^		
						Chinese (19.5)	Asian Indian (22.4)	Other Asian (58.2)
Sex
Male	1,205 (48.8)	949 (45.5)	479 (51.6)	361 (47.7)	471 (47.4)	(49.5)	(50.1)	(45.6)
Female	1,169 (51.2)	1,008 (54.5)	441 (48.4)	394 (52.3)	474 (52.6)	(50.5)	(49.9)	(54.4)
Age
6–11 years	242 (6.1)	320 (9.9)	222 (13.6)	114 (8.8)	89 (5.8)	(6.9)	(5.5)	(5.6)
12–19 years	251 (10.0)	346 (15.1)	199 (18.0)	125 (13.0)	155 (10.9)	(9.2)	(8.5)	(12.3)
20–39 years	622 (26.0)	415 (31.0)	213 (37.8)	161 (36.1)	293 (36.6)	(38.3)	(39.6)	(34.9)
40–59 years	571 (32.6)	454 (29.3)	180 (24.3)	168 (28.0)	252 (31.1)	(27.2)	(34.4)	(31.1)
≥ 60 years	688 (25.3)	422 (14.7)	106 (6.3)	187 (14.2)	156 (15.6)	(18.3)	(11.9)	(16.1)
Education
< High school (HS)	394 (11.5)	393 (18.6)	506 (51.8)	286 (35.3)	147 (14.1)	(8.3)	(11.9)	(16.8)
HS graduate/GED	488 (19.5)	520 (26.1)	186 (21.1)	167 (23.7)	127 (12.6)	(9.3)	(9.4)	(15.0)
Some college/AA	786 (33.0)	693 (37.5)	162 (19.7)	173 (24.0)	206 (22.2)	(19.7)	(16.6)	(25.3)
≥ College graduate	687 (36.0)	324 (17.7)	61 (7.4)	116 (17.0)	456 (51.1)	(62.7)	(62.0)	(42.9)
Household Income
< $20,000	557 (13.4)	584 (32.5)	240 (27.3)	207 (29.1)	112 (12.4)	(12.4)	(9.6)	(13.6)
$20,000–< $50,000	805 (31.7)	693 (37.2)	444 (48.8)	274 (38.8)	261 (30.8)	(29.0)	(24.6)	(34.0)
$50,000–< $75,000	220 (12.5)	165 (9.1)	82 (10.7)	78 (12.1)	94 (11.9)	(8.1)	(16.2)	(11.5)
≥ $75,000	703 (42.4)	374 (21.2)	104 (13.2)	133 (20.0)	364 (44.9)	(50.5)	(49.6)	(41.0)
Poverty to income ratio
≤ Median (1.63)	970 (25.8)	942 (51.9)	547 (61.5)	372 (53.6)	241 (26.4)	(22.1)	(17.2)	(31.5)
> Median (1.63)	1,299 (74.2)	843 (48.1)	291 (38.5)	312 (46.4)	584 (73.6)	(77.9)	(82.9)	(68.5)
Birthplace
USA	2,275 (96.1)	1,790 (91.4)	538 (53.7)	293 (36.9)	277 (24.8)	(27.3)	(15.9)	(27.4)
Outside USA	99 (3.9)	167 (8.6)	380 (46.3)	460 (63.1)	668 (75.2)	(72.7)	(84.1)	(72.7)
BMI
Underweight	52 (2.0)	42 (2.2)	14 (1.4)	13 (1.8)	42 (4.1)	(5.2)	(3.9)	(3.9)
Normal	859 (35.2)	660 (30.4)	321 (30.9)	254 (31.4)	569 (60.2)	(71.5)	(51.7)	(59.7)
Overweight	712 (32.5)	474 (25.1)	248 (29.1)	233 (32.8)	228 (25.2)	(17.5)	(30.0)	(26.0)
Obese	719 (30.3)	757 (42.3)	324 (38.5)	253 (34.1)	93 (10.4)	(5.8)	(14.4)	(10.4)
Smoking (cotinine level)^*c*^
1st tertile	846 (41.3)	364 (19.6)	371 (40.0)	314 (41.3)	339 (36.6)	(37.1)	(27.2)	(40.1)
2nd tertile	601 (26.2)	621 (32.4)	343 (37.0)	250 (33.4)	427 (45.9)	(48.1)	(59.3)	(40.0)
3rd tertile	873 (32.5)	896 (48.0)	183 (23.0)	174 (25.3)	160 (17.4)	(14.8)	(13.5)	(19.9)
Recent fish consumption^*d*^
Yes	1,490 (68.6)	1,277 (71.4)	472 (58.6)	430 (64.9)	605 (77.4)	(85.7)	(56.4)	(83.0)
No	774 (31.4)	543 (28.6)	374 (41.4)	247 (35.1)	189 (22.6)	(14.3)	(43.7)	(17.0)
Urbanization^*b*^
Metro center	(23.8)	(46.9)	(49.8)	(67.1)	(65.4)		*—*^*e*^
Metro fringe	(26.3)	(28.8)	(4.8)	(22.8)	(23.8)
Other	(49.9)	(24.3)	(45.3)	(10.0)	(10.8)
U.S. Census region^*b*^
Northeast	(14.3)	(11.7)	(5.1)	(36.3)	(25.0)
Midwest	(29.3)	(14.1)	(4.5)	(2.5)	(7.6)		*—*^*e*^
South	(30.8)	(67.3)	(39.0)	(46.2)	(28.0)
West	(25.6)	(6.9)	(51.4)	(15.1)	(39.5)
Abbreviations: AA, Associate in Art degree; GED, General Educational Development. ^***a***^Sample counts and weighted percentage among five NHANES race and ethnic groups and weighted percentage among three Asian subgroups. ^***b***^Raw sample counts are not provided for the restricted data. ^***c***^Cotinine levels: 1st tertile (< 0.019 ng/mL), 2nd tertile (0.019–< 0.144 ng/mL), 3rd tertile (≥ 0.144 ng/mL). ^***d***^Fish eaten during past 30 days. ^***e***^Because of potential disclosure risk, geographical analysis on Asian subgroups is not included.								

### Statistical Analysis

All statistical analyses were performed using SAS-callable SUDAAN version 11.0.1 (RTI International, Research Triangle Park, NC, USA) installed as an add-on to SAS software version 9.3 or higher (SAS Institute Inc., Cary, NC, USA). We accounted for the NHANES’s complex sample design and applied appropriate strata, cluster, and weights, as described in the NHANES documentation (CDC 2015), in all the statistical analyses.

We stratified the data by five NHANES race/ethnic groups: non-Hispanic white, non-Hispanic black, Mexican American, other Hispanic and Asian subgroups (Chinese, Asian Indian, and Other Asian), and computed weighted statistics for biomarker levels by each covariate. The statistics included the geometric mean and its 95% confidence interval (CI), as well as the 50th and 95th percentiles based on the Taylor series linearization method (RTI International 2012). Summary statistics were presented for five biomarker variables [B-Cd, B-Pb, B-Hg, U-TAs (creatinine-corrected), and U-DMA (creatinine-corrected)]. In accordance with the *Fourth National Report on Human Exposure to Environmental Chemicals*, the geometric mean concentration was not calculated when the level for a biomarker was below the LOD in > 40% of the samples (CDC 2014). For the protection of study participants’ confidentiality, analyses using geographical covariates (urbanization and census region) were not conducted for Asian subgroups.

We compared geometric means of biomarker levels for each covariate category across five NHANES race/ethnic groups and then compared geometric means of biomarker levels across three Asian subgroups, using analysis of variance (ANOVA). Further, differences in geometric means within each covariate were assessed using ANOVA, stratified by NHANES race/ethnic group and Asian subgroup. For all analyses, *p* < 0.05 was considered statistically significant.

## Results

### Sample Characteristics

Table 1 presents the study participants’ characteristics by racial/ethnic group. The final number of samples included in the analysis was 6,951 out of 9,756; approximately one-third (2,427) were used for urinary biomarker analyses.

Since differences in biomarker levels may reflect group characteristics such as socioeconomic status and dietary patterns, we first examined the comparability of the various racial/ethnic groups and subgroups by the covariates. The distribution of age groups varied across the racial/ethnic groups. The Asian group had a distribution similar to those of blacks and other Hispanics and tended to be younger than whites and older than Mexican Americans. The Asian group had the highest percentage of college graduates or above. Socioeconomic status (denoting household income and PIR) of the Asian group mirrored that of the white group, with these two groups having higher percentages of the highest income category (> $75,000) and above median PIR than the other three groups. Asians had the lowest percentage of U.S.-born participants (24.8%), compared with > 90% of the white and black populations having been born in the United States. The distributions of recent fish consumers (those who had eaten fish in the 30 days before the study) were generally comparable across the five groups. Large geographical variations existed across the groups. Asians as well as Hispanics and Mexican Americans tended to live in urban areas, with the largest populations of Asian and Mexican-American participants being found in the West.

The weighted percentages of the Asian subgroup samples (Chinese and Asian Indians) were roughly proportional to those observed in the 2010 U.S. Census data (Hoeffel et al. 2012). In general, age groups were distributed similarly. Education and economic status among Chinese and Asian Indians was higher than those of Other Asians. Asian Indians had an approximately 10% lower percentage of U.S.-born individuals than other two subgroups. The proportion of individuals with a normal BMI was highest among Chinese. There was a noticeably higher rate of recent fish consumers in the Chinese and Other Asian subgroups (> 80%) than that of Asian Indians (56.4%).

### Analysis of Biomarker Data

Weighted summary statistics of biomarker data (geometric mean and 50th and 95th percentile) are provided in Tables S1–S5 for the five groups and in Tables S6–S10 for the three Asian subgroups.

### Overall Comparison across Racial/Ethnic Groups and Asian Subgroups

For all biomarkers, the geometric mean value in Asians was significantly (*p* < 0.05) higher than that in each of the other racial/ethnic groups (Table 2). This observation was consistent in nearly all of the comparisons performed within subsets of data based on the various demographic, socioeconomic, physical, dietary, behavioral, and geographical characteristics. Biomarker levels among Asians were significantly lower than those of other groups in only two cases: the comparisons of B-Cd and B-Pb levels in U.S.-born individuals (see Tables S1 and S2). For all other comparisons, biomarker levels among Asians are either the highest (mostly significantly) or not significantly different from those of other race/ethnic groups with higher biomarker levels.

**Table 2:** Comparison of weighted geometric mean biomarker levels across NHANES racial and ethnic group.

Group	Cadmium (blood) (μg/L)			Lead (blood) (μg/dL)		Mercury (blood) (μg/L)		Arsenic, total (urinary) (μg/g-creatinine)			DMA (urinary) (μg/g-creatinine)		
	*n*^*a*^	GM (95% CI)	*p*-Value	GM (95% CI)	*p*-Value	GM (95% CI)	*p*-Value	*n*	GM (95% CI)	*p*-Value	*n*	GM (95% CI)	*p*-Value
Non-Hispanic Asian^*b*^	945	0.41 (0.37, 0.45)		1.16 (1.07, 1.25)		1.93 (1.65, 2.27)		353	22.3 (19.1, 26.1)		356	9.89 (8.58, 11.41)
Non-Hispanic white	2,374	0.29 (0.27, 0.31)	< 0.001	1.00 (0.92, 1.08)	0.004	0.71 (0.61, 0.84)	< 0.001	818	7.13 (6.05, 8.39)	< 0.001	824	3.68 (3.44, 3.93)	< 0.001
Non-Hispanic black	1,957	0.31 (0.29, 0.33)	< 0.001	0.98 (0.93, 1.03)	0.002	0.71 (0.57, 0.89)	< 0.001	669	7.24 (5.53, 9.48)	< 0.001	672	3.16 (2.67, 3.73)	< 0.001
Mexican American	920	0.23 (0.21, 0.24)	< 0.001	0.83 (0.76, 0.91)	< 0.001	0.51 (0.45, 0.58)	< 0.001	317	8.00 (6.87, 9.32)	< 0.001	317	4.12 (3.84, 4.43)	< 0.001
Other Hispanic	755	0.25 (0.23, 0.28)	< 0.001	0.88 (0.79, 0.98)	< 0.001	0.91 (0.81, 1.02)	< 0.001	256	9.25 (8.17, 10.49)	< 0.001	257	5.02 (4.50, 5.61)	< 0.001
DMA, dimethylarsinic acid. ^***a***^Sample size was the same for all three blood biomarkers (cadmium, lead, and mercury). ^***b***^Asians were used as the reference group.													

Across the Asian subgroups, biomarker levels were generally similar between the Chinese and Other Asian subgroups (Table 3). The Asian-Indian subgroup had lower biomarker levels than those of the other two Asian subgroups, with the exception of B-Pb. Although the differences in B-Pb levels were not significant, Asian Indians had the highest overall geometric mean B-Pb across the three Asian subgroups. In comparisons made within Asian subgroups, B-Pb levels were significantly higher among Asian Indians for adolescents (12–19 years old) (0.90 μg/dL), older adults (≥ 60 years old) (2.19 μg/dL), those with household income ≥ $75,000 (1.33 μg/dL), and above-mean PIR (1.37 μg/dL) categories than those in the other two Asian subgroups.

**Table 3:** Comparison of weighted geometric mean biomarker levels across Asian subgroup.

Covariate	Cadmium (blood) (μg/L)				Lead (blood) (μg/dL)				Mercury (blood) (μg/L)				Arsenic, total (urinary) (μg/g-creatinine)				DMA (urinary) (μg/g-creatinine)			
	C	AI	Other	(^*a*^)	C	AI	Other	(^*a*^)	C	AI	Other	(^*a*^)	C	AI	Other	(^*a*^)	C	AI	Other	(^*a*^)
Overall	0.45	0.31	0.43	< 0.001	1.22	1.29	1.10	0.112	2.58	0.79	2.48	< 0.001	23.07	10.94	28.18	< 0.001	9.84	6.27	11.56	< 0.001
Sex
Male	0.42	0.28	0.34	< 0.001	1.41	1.45	1.22	0.203	2.70	0.86	2.48	< 0.001	26.30	8.83	23.82	< 0.001	9.71	4.99	10.07	< 0.001
Female	0.49	0.34	0.53	< 0.001	1.06	1.15	1.00	0.110	2.47	0.73	2.48	< 0.001	19.19	14.00	31.92	< 0.001	10.05	8.13	12.82	0.010
(*p*-Value^*b*^)	0.10	0.03	< 0.001		< 0.001	0.002	0.003		0.39	0.28	1.00		0.11	0.01	0.07		0.83	< 0.001	0.08
Age
6–11 years	0.20	0.14	0.14	0.056	0.74	0.64	0.91	0.160	1.05	0.78	0.72	0.243	32.66	13.58	17.87	0.152	13.6	10.9	10.87	0.693
12–19 years	0.20	0.22	0.21	0.909	0.63	0.90	0.69	< 0.001	1.42	0.78	1.11	0.052	11.17	6.81	11.32	0.037	6.15	3.88	6.41	0.006
20–39 years	0.46	0.29	0.42	< 0.001	1.23	1.06	0.92	0.161	2.23	0.60	2.18	< 0.001	20.85	11.32	24.21	0.003	8.34	6.19	9.57	0.054
40–59 years	0.57	0.38	0.55	< 0.001	1.49	1.65	1.39	0.328	3.98	1.18	3.93	< 0.001	30.06	12.94	37.50	< 0.001	10.97	7.38	14.03	0.006
≥ 60 years	0.62	0.41	0.74	0.005	1.52	2.19	1.53	0.003	3.52	0.62	3.80	< 0.001	24.04	8.04	52.85	< 0.001	12.79	4.68	18.37	< 0.001
(*p*-Value^*b*^)	< 0.001	< 0.001	< 0.001		< 0.001	< 0.001	< 0.001		< 0.001	0.07	< 0.001		0.03	0.04	< 0.001		0.12	< 0.001	< 0.001
Education
< High school (HS)	0.55	0.38	0.60	0.078	1.39	1.90	1.46	0.108	2.84	0.64	2.80	0.013	18.54	10.14	38.28	0.007	9.20	5.59	17.62	0.001
HS graduate/GED	0.64	0.29	0.47	< 0.001	1.42	1.10	1.29	0.455	2.80	3.40	3.08	0.543	32.66	16.56	21.23	0.323	11.97	8.32	10.23	0.311
Some college/AA	0.52	0.34	0.39	0.001	1.45	1.04	1.02	0.038	2.89	0.74	2.33	< 0.001	25.29	8.63	28.51	0.035	10.99	5.16	10.81	0.226
≥ College graduate	0.40	0.29	0.41	< 0.001	1.13	1.30	0.96	0.002	2.46	0.67	2.32	< 0.001	20.46	10.91	27.10	< 0.001	8.81	6.40	10.23	< 0.001
(*p*-Value^*b*^)	< 0.001	0.52	0.003		0.07	0.03	< 0.001		0.81	< 0.001	0.40		0.44	0.36	0.11		0.50	0.26	0.13
Household income
< $20,000	0.65	0.30	0.61	0.006	1.38	0.82	1.20	0.153	2.74	1.06	3.35	0.023	39.81	16.90	38.46	0.027	15.96	8.28	17.54	0.002
$20,000–< $50,000	0.60	0.32	0.45	< 0.001	1.49	1.45	1.10	0.013	2.45	0.58	2.18	< 0.001	27.99^*c*^	9.44^*c*^	23.34^*c*^	< 0.001	10.59^*c*^	5.90^*c*^	10.40^*c*^	0.003
$50,000–< $75,000	0.40	0.35	0.44	0.572	1.19	1.38	1.07	0.206	1.77	0.85	2.22	0.036
≥ $75,000	0.35	0.30	0.37	0.012	1.06	1.33	1.04	0.022	2.72	0.63	2.64	< 0.001	17.58	9.51	27.93	< 0.001	8.19	5.75	10.94	0.001
(*p*-Value^*b*^)	< 0.001	0.86	0.001		0.07	0.14	0.45		0.12	0.25	0.26		< 0.001	0.25	0.11		0.03	0.55	0.13
Poverty to income ratio	
≤ Median (1.63)	0.64	0.34	0.49	0.014	1.48	1.06	1.19	0.380	2.87	0.86	2.81	0.034	39.36	14.29	30.27	0.055	13.77	7.02	14.56	0.013
> Median (1.63)	0.40	0.31	0.40	< 0.001	1.15	1.37	1.03	< 0.001	2.50	0.65	2.36	< 0.001	18.75	9.68	25.82	< 0.001	8.59	5.93	10.40	< 0.001
(*p*-Value^*b*^)	0.02	0.54	0.01		0.13	0.19	0.12		0.51	0.53	0.34		< 0.001	0.31	0.36		0.02	0.63	0.05
Birthplace
USA	0.27	0.17	0.25	0.002	0.82	0.71	0.79	0.637	2.25	0.88	1.51	< 0.001	17.14	9.04	16.52	0.009	8.19	5.07	7.85	0.003
Outside USA	0.55	0.35	0.53	< 0.001	1.42	1.44	1.24	0.151	2.72	0.77	2.99	< 0.001	25.24	11.35	33.73	< 0.001	10.45	6.53	13.18	< 0.001
(*p*-Value^*b*^)	< 0.001	< 0.001	< 0.001		< 0.001	< 0.001	< 0.001		0.38	0.49	< 0.001		0.16	0.28	< 0.001		0.26	0.14	< 0.001
BMI
Underweight	0.57	0.21	0.47	0.003	1.33	0.65	1.20	0.092	2.72	0.40	1.75	< 0.001	—^*d*^				—^*d*^		
Normal	0.45	0.29	0.43	< 0.001	1.14	1.29	1.09	0.112	2.38	0.79	2.56	< 0.001	21.04	10.79	31.05	< 0.001	9.16	5.93	12.30	< 0.001
Overweight	0.44	0.34	0.47	0.006	1.45	1.41	1.15	0.085	3.31	0.89	2.86	< 0.001	32.14	11.38	25.70	< 0.001	12.82	6.70	10.67	0.014
Obese	0.41	0.34	0.37	0.604	1.58	1.26	0.99	0.009	3.75	0.76	1.73	< 0.001	—^*d*^				—^*d*^		
(*p*-Value^*b*^)	0.43	0.10	0.33		< 0.001	0.07	0.36		0.01	0.15	0.04
Smoking (cotinine level)	
1st tertile	0.35	0.24	0.36	< 0.001	1.02	0.87	0.94	0.541	2.34	0.99	2.42	< 0.001	23.71	13.06	27.16	< 0.001	10.16	6.73	11.80	< 0.001
2nd tertile	0.46	0.31	0.45	< 0.001	1.25	1.46	1.22	0.099	2.77	0.65	2.61	< 0.001	20.85	10.16	27.86	< 0.001	9.16	6.31	11.40	0.008
3rd tertile	0.76	0.56	0.60	0.314	1.77	1.69	1.24	0.180	2.66	1.10	2.32	0.002	25.53	8.93	29.58	< 0.001	11.02	4.22	10.12	< 0.001
(*p*-Value^*b*^)	< 0.001	< 0.001	< 0.001		0.02	0.01	0.004		0.68	0.13	0.62		0.71	0.35	0.95		0.59	0.02	0.80
Recent fish consumption	
Yes	0.43	0.28	0.44	< 0.001	1.20	1.22	1.11	0.427	2.71	1.71	2.88	< 0.001	21.09	15.07	29.85	0.001	9.25	7.41	11.12	0.017
No	0.35	0.33	0.28	0.524	1.01	1.31	0.83	< 0.001	1.17	0.30	0.74	< 0.001	8.71	7.96	10.16	0.558	4.67	5.19	6.46	0.475
(*p*-Value^*b*^)	0.27	0.09	< 0.001		0.22	0.50	< 0.001		0.003	< 0.001	< 0.001		0.01	0.002	< 0.001		0.01	0.03	< 0.001
Abbreviations: AA, Associate in Art (AA) degree; AI, Asian Indian; C, Chinese; GED, General Educational Development. ^***a***^Significance of difference in geometric mean across Asian subgroups. ^***b***^Significance of difference in geometric mean across categories within covariate. ^***c***^Due to small sample size, the results for two income ranges ($20,000–< $50,000 and $50,000–< $75,000) were aggregated. ^***d***^Results are not presented due to small sample size.																				

### Predictors of Biomarker Levels in Asian Subgroups

***Cadmium.*** Sex was significantly associated with B-Cd levels in two of the three Asian subgroups. Females had higher B-Cd levels than males across all subgroups (Table 3). A general trend of increasing B-Cd with age was observed. There was an apparent inverse trend with socioeconomic status (education, income, and PIR) and B-Cd levels. B-Cd levels were significantly higher in individuals born outside of the United States, compared with those born in the United States in all of the Asian subgroups. A clear trend of B-Cd levels increasing with cotinine levels was observed in all subgroups.

***Lead.*** B-Pb levels were significantly associated with sex. B-Pb levels were significantly higher among males than females in all three Asian subgroups (Table 3). B-Pb level generally increased with age. There was a general trend of decreasing B-Pb levels with higher educational status. Individuals born outside of the United States had higher B-Pb levels than those born in the United States across all of the Asian subgroups. A clear trend of B-Pb levels increasing with cotinine levels was observed in all subgroups.

***Mercury.*** A general trend of increasing B-Hg levels with age was observed, with the exception of the Asian-Indian subgroup (Table 3). Significant differences in B-Hg across BMI categories were observed among Chinese and Other Asian subgroups, although no consistent pattern of B-Hg was seen between these two subgroups. Recent fish consumers had higher B-Hg levels than non-consumers in all three Asian subgroups.

***Arsenic, total.*** The general patterns of the U-tAs levels across age groups were similar in all Asian subgroups (Table 3). U-tAs levels decreased from the youngest group (6–11 years) to the second youngest age group (12–19 years) and then generally increased with age after childhood (≥ 12 years). U-tAs levels were significantly higher among recent fish consumers than non-consumers in all three Asian subgroups.

***DMA.*** The patterns of the U-DMA levels across age groups were similar to those of the U-tAs (Table 3). U-DMA levels were often higher among the youngest age group (6–11 years) than those among other age groups. Across the age groups (≥ 12 years), there was a general trend of increasing U-DMA levels with age. Recent fish consumers had higher U-DMA levels than non-consumers in all three Asian subgroups.

## Discussion

Our study confirmed there are racial/ethnic differences in the biomarker levels of toxic metals—cadmium, lead, mercury, and arsenic—in the United States. Overall, biomarker levels among Asians were higher than in other racial/ethnic groups regardless of sociodemographic, physical, behavioral, dietary, and geographic characteristics (see Tables S1–S5). Asians had significantly lower biomarker levels than other groups in only two comparisons: *a*) The B-Cd among U.S.-born blacks was significantly higher than that among U.S.-born Asians, and *b*) U.S.-born whites and blacks had significantly higher B-Pb levels than U.S.-born Asians. Across the Asian subgroups, the lowest biomarker levels were generally observed among Asian Indians, except for B-Pb levels. Although no significant difference was observed in the overall comparison of B-Pb levels across Asian subgroups (≥ 6 years old), significantly higher B-Pb levels among Asian Indians were found in adolescents (12–19 years old), older adults (≥ 60 years old), people in the highest income category (≥ $75,000), and people above the median PIR. The elevated B-Pb levels in Asian Indians may be associated with their spice and cosmetic use, since elevated levels of lead have been found in turmeric (Gleason et al. 2014), a main ingredient of curry, and in eye makeup, such as surma or kohl, that are often used in Indian communities (Goswami 2013).

In general, biomarker levels among Asians in the United States were lower than the levels reported in studies conducted in Asian countries. Ding et al. (2014) evaluated the B-Cd and B-Pb levels of the general population in China, based on randomly selected study participants aged 6–60 years old (*n* = 18,120) from 24 districts in eight provinces in China between 2009 and 2010. Geometric mean B-Cd and B-Pb levels from this study were 0.49 μg/L and 3.49 μg/dL, respectively, compared with the geometric mean B-Cd (0.45 μg/L) and B-Pb (1.22 μg/L) levels observed among the Chinese subgroup in the present study (Table 4). Geometric mean blood biomarker levels (2011) reported in the Korea NHANES (Seo et al. 2015), a Korean national health survey similar to the CDC’s NHANES, were slightly higher, but comparable with the levels observed among the Other Asian subgroup, which is assumed to consist mainly of Filipino, Vietnamese, Korean, and Japanese according to the 2010 Census (Hoeffel et al. 2012). The geometric mean blood biomarker levels among those Koreans ≥ 19 years were 0.86 μg/L (B-Cd), 1.99 μg/dL (B-Pb), and 3.08 μg/L (B-Hg) (Table 4). In our study, the ranges of the geometric mean of B-Cd, B-Pb, and B-Hg levels in the corresponding age group (≥ 20 years old) of Other Asians were 0.42–0.74 μg/L (B-Cd), 0.92–1.53 μg/dL (B-Pb), and 2.18–3.80 μg/L (B-Hg). Urinary arsenic levels in Koreans were noticeably higher than the levels observed in the present study. Geometric mean U-tAs levels reported in the Korea NHANES (2008–2009) ranged from 90.6 μg/g-creatinine (20–39 years old) to 157.6 μg/g-creatinine (≥ 60 years old) (Rhee et al. 2013), whereas U-tAs levels observed in our study were 24.21 μg/g-creatinine (20–39 years old) to 52.85 μg/g-creatinine (≥ 60 years old) among the Other Asian subgroup (Table 4).

**Table 4:** Comparison of geometric mean biomarker levels of Asian subgroups in the U.S. to those reported in Asian countries.

Metal	NHANES 2011–2012			Studies in Asian countries			
	Subgroup	Concentration	Age group	Country	Concentration	Age group	Reference
Cadmium (μg/L)	Chinese	0.45	≥ 6 years	China	0.49	6–60 years	Ding et al. 2014
	Other Asian	0.42–0.74	≥ 20 years	Korea	0.86	≥ 19 years	Seo et al. 2015
Lead (μg/dL)	Chinese	1.22	≥ 6 years	China	3.49	6–60 years	Ding et al. 2014
	Other Asian	0.92–1.53	≥ 20 years	Korea	1.99	≥ 19 years	Seo et al. 2015
Mercury (μg/L)	Other Asian	2.18–3.80	≥ 20 years	Korea	3.08	≥ 19 years	Seo et al. 2015
Arsenic, total (μg/g-creatinine)	Other Asian	24.2–52.8	≥ 20 years	Korea	90.6–157.6	≥ 20 years	Rhee et al. 2013

Except for lead, the exposure pathway of the metals we evaluated is known to be predominantly food consumption for the general population. Seafood is the major source of dietary exposure to mercury (methylmercury) and arsenic (total) (ATSDR 1999, 2007a). In addition to seafood, cereal grains (including rice) and poultry are the major contributors to dietary arsenic exposure in the United States (Tsuji et al. 2007; Vogt et al. 2012; Xue et al. 2010). Smoking is the main source of cadmium exposure (ATSDR 2012), though exposure to cadmium for nonsmokers occurs mostly through diet, such as consumption of vegetables and cereal grains (Egan et al. 2007; He et al. 2013). Sources of lead exposure include environmental exposure through lead-containing dust and soil from hazardous waste sites, highways, and old fruit orchards; smoking; drinking water from old plumbing systems; inhalation or direct contact with lead-based paint; and ingestion of food from lead-glazed potteries or dishes (ATSDR 2007b). In this study, recent fish consumption was a significant predictor for B-Hg and U-tAs levels. In addition, positive dose–response relationships were found for cotinine levels (an indicator of smoking) and both B-Cd and B-Pb in each of the Asian subgroups.

Further, our study found that several other characteristics are important predictors of biomarker levels. Sex and age differences in biomarker levels were generally consistent across Asian subgroups. Females had higher B-Cd and lower B-Pb levels than males. Biomarker levels generally increased with age. A higher level of U-tAs and U-DMA were observed in the youngest age group (6–11 years). This may be attributable to greater arsenic exposure and/or age-dependent toxicokinetc characteristics (e.g., efficient absorption or poor excretion of arsenic) of this age group. Additionally, we found birthplace to be an important predictor of biomarker levels: consistently higher biomarker levels (albeit not always significant) were observed among Asians born outside of the United States compared with Asians born in the United States. Although higher, the biomarker levels among non–U.S.-born Asians are less than the levels reported in their countries of origins described in the previous paragraph. Further, as discussed earlier, within the comparisons among U.S.-born individuals, Asians had significantly lower B-Cd and B-Pb than those of other racial/ethnic groups. A further characterization of metal exposure depending on birthplace and its relationship with biomarker levels will be warranted in future studies. These patterns of biomarker levels based on sex, age, and birthplace among Asians agreed with the results reported in previous studies based on the general U.S. population (Caldwell et al. 2009; Mortensen et al. 2014; Peters et al. 2014). In contrast, there appear to be different patterns of B-Hg and U-tAs among Asians for the covariates representing socioeconomic status. A general trend of increasing B-Hg and U-tAs with increasing educational and socioeconomic status was observed among the racial/ethnic groups other than Asians, with this trend being more pronounced in the white group. This result was consistent with the results of previous studies (Buchanan et al. 2015; McKelvey et al. 2007; Mortensen et al. 2014). It is typically explained that individuals with higher incomes and/or educational achievement can afford to add larger fish (e.g., tuna, swordfish), which tend to have higher mercury content, to their diet (Hightower and Moore 2003; Mortensen et al. 2014). However, this trend was reversed among the Asian population. One possible explanation for this difference is that Asians of lower socioeconomic status may consume fish containing higher levels of mercury. For example, some economically disadvantaged Asian subgroups may be more likely to engage in subsistence fishing and consume locally harvested fish that have higher levels of environmental contaminants.

There are several limitations associated with the present study. First, because of the cross-sectional design of the NHANES, the data represent only a snapshot of biomarker levels on the day of examination. Similarly, some of the covariates (smoking based on cotinine levels, and fish consumption) only reflect the participants’ living environment or food consumption patterns immediately before the survey, and may not represent their long-term exposure. Second, the toxic metals evaluated in this study have different half-lives in the human body. Cadmium is not readily excreted and has a long biological half-life (as long as 38 years) (ATSDR 2008). Although the biological half-life of lead is approximately 30 days, it tends to accumulate in the bones and soft tissues over a long time and is released very slowly (ATSDR 2010). Mercury, predominantly present in the blood as methylmercury, has a half-life of approximately 2 months (ATSDR 1999). Therefore, the blood biomarkers for cadmium and lead may be indicators suitable for the body burden after long-term exposure. The biological half-life of arsenic is fairly short, roughly 2–3 days (ATSDR 2007a). Because urinary biomarkers have short half-lives and reflect short-term exposure, they tend to vary more depending on the study participants’ food consumption, living environment, and occupational exposure immediately before the sampling. Also, because the information related to fish consumption is self-reported, it is subject to recall bias. Further, following the CDC’s analytical approach, urinary biomarker data corrected using urinary creatinine level were used in our study. Urinary creatinine levels vary depending on various factors such as sex, muscle mass, diet, and health conditions. Our supplemental comparisons (Tables S1–S10) of urinary creatinine levels across the racial/ethnic groups indicate lower levels of urinary creatinine among Asians than the other groups. These differences are also attributable to the higher U-tAs and U-DMA among Asians observed in this study. Our analysis evaluated the association between biomarker levels and a limited number of covariates representing study participants’ demographic, socioeconomic, physical, behavioral, and dietary characteristics. Covariates characterizing food consumption patterns were limited to fish intake; we did not include other important food sources of metal exposures. For instance, a significant association between biomarker levels of arsenic (both total and inorganic) and rice consumption has been reported (Davis et al. 2012; Wei et al. 2014); we did not analyze this. Further, we did not include covariates representing study participants’ living environment or occupational exposure in our study. A recent study based on NHANES data suggests that occupation is a significant predictor of blood lead and blood cadmium levels (Peters et al. 2014). Lead paint and use of lead-containing pottery may be important sources of environmental lead exposure. Inclusion of these covariates may have improved our characterization of metal exposure. Furthermore, there may be race/ethnicity specific differences in frequency of genetic variants that influence absorption, distribution, metabolism, elimination/excretion processes and such differences could also be related to differences in biomarker levels of metals across groups.

Another uncertainty associated with the current study is how representative our sample was of the Asian population. Asians typically have a lower participation rate in national surveys than other racial/ethnic groups, and the NHANES response rate among Asian in 2011 was approximately 10–20% lower than that of other groups (Broitman 2012). Because of potential response bias, the NCHS performed an analysis of nonresponders by comparing the demographic and socioeconomic characteristics of responders and nonresponders (NCHS 2013). Based on this analysis, the NCHS concluded that, although a potential for nonresponse bias may exist, weight adjustment lessens the bias. Our analysis used appropriate sample weights; however, it still remains uncertain to what extent this potential bias may have remained and distorted the results. Furthermore, we used biomarker levels of Asians from one NHANES data cycle. The Asian group was divided into three subgroups, and the results are based on a relatively small number of samples. Therefore, some of our results may be statistically unreliable and should be viewed with caution. Because oversampling of the Asian population continues in the next NHANES data cycle (2013–2014), the findings of this study should be verified with the larger data set in future studies.

This study also had several strengths. We evaluated differences in biomarker levels of five metals across different racial/ethnic groups in the United States, with a specific interest in the Asian population, due to previously reported elevated concentrations of metal biomarkers in this group. The NHANES 2011–2012 is the first data cycle to include a specific Asian race category, and to the best of our knowledge, this is one of the first studies to investigate biomarker levels in this historically less-studied racial group using nationally representative data. We evaluated biomarker levels of three subgroups of the Asian population: Chinese, Asian Indian, and Other Asian. Although NHANES is not designed to evaluate small sample groups and the results are not nationally representative, our study was able to assess general biomarker patterns among subgroups of Asians, which have rarely been evaluated, especially on a national scale.

According to the 2010 U.S. Census (U.S. Census Bureau 2013), Asians were the fastest-growing race/ethnic group in the United States with an increase of 43.2% between 2000 and 2010. As this study demonstrated, there are considerable variations in sociodemographic, behavioral, and exposure characteristics between Asians and other racial/ethnic groups and also between Asian subgroups. As the Asian population in the United States continues to grow, more studies are warranted to improve our understanding of the health and nutritional status of this minority group.

## Conclusion

Asian populations were found to have the highest levels of B-Cd, B-Pb, B-Hg, U-tAs, and U-DMA across the five racial/ethnic groups assessed in the NHANES. Generally, this observation did not change when data were further examined by various demographic, socioeconomic, physical, dietary, behavioral, and geographical characteristics. Within the Asian group, considerable variations in biomarker levels are present across the Chinese, Asian Indian, and Other Asian subgroups. Biomarker levels of toxic metals, except B-Pb, are generally lowest among Asian Indians. Sex, age, education, birthplace, smoking, and fish consumption were found to be significant predictors of biomarker levels for certain metals.

## Supplemental Material

(229 KB) PDF
