# Supplementary material for: Biomarker Levels of Toxic Metals among Asian Populations in the United States: NHANES 2011–2012
Source: Environ Health Perspect. 2016 Aug 12;125(3):306–13. doi: 10.1289/EHP27 (PMC5332180; doi:10.1289/EHP27)
Supplement: (229 KB) PDF [file EHP27.s001.acco.pdf]

Environ Health Perspect
DOI: 10.1289/EHP27

Note to readers with disabilities: *EHP* strives to ensure that all journal content is accessible to all readers. However, some figures and Supplemental Material published in *EHP* articles may not conform to 508 standards due to the complexity of the information being presented. If you need assistance accessing journal content, please contact ehp508@niehs.nih.gov. Our staff will work with you to assess and meet your accessibility needs within 3 working days.

Supplemental Material

Biomarker Levels of Toxic Metals among Asian Populations in the U.S.: NHANES 2011–2012

Hiroshi Awata, Stephen Linder, Laura E. Mitchell, and George L. Delclos

Table of Contents

LIST OF TABLES

Supplemental Material 1: Weighted statistics of biomarker levels by NHANES racial and ethnic

Table S1. Weighted statistics of blood cadmium levels (µg/L) by NHANES racial and ethnic group

Table S2. Weighted statistics of blood lead levels (µg/dL) by NHANES racial and ethnic group

Table S3. Weighted statistics of blood mercury (total) levels (µg/L) by NHANES racial and ethnic group

Table S4. Weighted statistics of urinary arsenic (total) levels (µg/g-creatinine) by NHANES racial and ethnic group

Table S5. Weighted statistics of urinary dimethylarsonic acid (DMA) levels (µg/g-creatinine) by NHANES racial and ethnic group

Supplemental Material 2: Weighted statistics of biomarker levels by Asian subgroup

Table S6. Weighted statistics of blood cadmium levels (µg/L) by Asian subgroup

Table S7. Weighted statistics of blood lead levels (µg/dL) by Asian subgroup

Table S8. Weighted statistics of blood mercury (total) levels (µg/L) by Asian subgroup

Table S9. Weighted statistics of urinary arsenic (total) levels (µg/g-creatinine) by Asian subgroup

Table S10. Weighted statistics of urinary dimethylarsonic acid (DMA) levels (µg/g-creatinine) by Asian subgroup

Supplemental Material 1: Weighted statistics of biomarker levels by NHANES racial and ethnic group

Table S1. Weighted statistics of blood cadmium levels (μg/L) by NHANES racial and ethnic group

	Non-Hispanic White			Non-Hispanic Black			Mexican American			Other Hispanic			Non-Hispanic Asian			p-value [b]
	N, Geometric Mean (95% CI)	Percentile 50th	Percentile 95th	N, Geometric Mean (95% CI)	Percentile 50th	Percentile 95th	N, Geometric Mean (95% CI)	Percentile 50th	Percentile 95th	N, Geometric Mean (95% CI)	Percentile 50th	Percentile 95th	N, Geometric Mean (95% CI)	Percentile 50th	Percentile 95th	
Overall	2374, 0.29 (0.27-0.31)	0.25	1.68	1957, 0.31 (0.29-0.33)	0.27	1.50	920, 0.23 (0.21-0.24)	0.21	0.86	755, 0.25 (0.23-0.28)	0.23	1.06	945, 0.41 (0.37-0.45)	0.39	1.38	<.001
Sex																
Male	1205, 0.27 (0.24-0.30)	0.21	1.63	949, 0.30 (0.27-0.32)	0.24	1.54	479, 0.21 (0.20-0.23)	0.19	0.91	361, 0.23 (0.21-0.26)	0.20	1.10	471, 0.34 (0.29-0.39)	0.32	1.37	<.001
Female	1169, 0.32 (0.30-0.35)	0.29	1.70	1008, 0.32 (0.29-0.34)	0.29	1.47	441, 0.24 (0.23-0.26)	0.23	0.81	394, 0.27 (0.24-0.31)	0.26	0.93	474, 0.48 (0.44-0.52)	0.47	1.40	<.001
p-value [a]	0.009			0.237			<.001			0.008			<.001			
Age																
6-11 yrs.	242, †	†	0.21	320, 0.13 (0.13-0.14)	†	0.25	222, †	†	0.23	114, 0.14 (0.12-0.16)	†	0.27	89, 0.15 (0.14-0.17)	†	0.33	0.046
12-19 yrs.	251, 0.17 (0.16-0.18)	†	0.66	346, 0.19 (0.17-0.21)	0.17	0.78	199, 0.16 (0.15-0.18)	0.13	0.33	125, 0.18 (0.15-0.21)	0.15	0.44	155, 0.21 (0.18-0.24)	0.21	0.57	0.010
20-39 yrs.	622, 0.27 (0.23-0.30)	0.22	1.59	415, 0.33 (0.29-0.37)	0.27	1.55	213, 0.25 (0.22-0.28)	0.24	0.75	161, 0.25 (0.22-0.30)	0.22	1.13	293, 0.39 (0.35-0.44)	0.37	1.39	<.001
40-59 yrs.	571, 0.35 (0.33-0.38)	0.29	2.00	454, 0.43 (0.39-0.47)	0.37	1.80	180, 0.31 (0.28-0.35)	0.29	1.04	168, 0.29 (0.26-0.33)	0.27	0.95	252, 0.50 (0.45-0.56)	0.48	1.41	<.001
60+ yrs.	688, 0.39 (0.36-0.42)	0.37	1.47	422, 0.42 (0.38-0.46)	0.40	1.42	106, 0.36 (0.31-0.42)	0.33	0.92	187, 0.39 (0.34-0.44)	0.37	1.34	156, 0.64 (0.55-0.74)	0.66	1.49	<.001
p-value [a]	<.001			<.001			<.001			<.001			<.001			
Education																
<High school (HS)	394, 0.43 (0.36-0.50)	0.40	2.16	393, 0.37 (0.34-0.40)	0.33	1.61	506, 0.23 (0.21-0.25)	0.21	0.82	286, 0.26 (0.23-0.31)	0.24	0.98	147, 0.54 (0.45-0.65)	0.56	1.71	<.001
HS graduate/GED	488, 0.31 (0.27-0.35)	0.26	1.79	520, 0.34 (0.30-0.38)	0.29	1.60	186, 0.23 (0.20-0.27)	0.21	0.94	167, 0.25 (0.23-0.28)	0.23	1.05	127, 0.45 (0.36-0.56)	0.49	1.19	<.001
Some college/AA	786, 0.32 (0.28-0.35)	0.27	1.80	693, 0.30 (0.27-0.32)	0.26	1.47	162, 0.23 (0.20-0.27)	0.23	0.77	173, 0.27 (0.23-0.32)	0.25	1.14	206, 0.40 (0.35-0.46)	0.40	1.50	<.001
≥College graduate	687, 0.24 (0.22-0.25)	0.22	0.82	324, 0.25 (0.23-0.28)	0.24	0.99	61, 0.20 (0.17-0.24)	0.18	0.53	116, 0.22 (0.19-0.26)	0.19	0.87	456, 0.37 (0.34-0.41)	0.36	1.19	<.001
p-value [a]	<.001			<.001			0.358			0.012			<.001			
Household Income																
<$20,000	557, 0.37 (0.29-0.48)	0.32	2.09	584, 0.35 (0.32-0.38)	0.30	1.66	240, 0.22 (0.20-0.23)	0.19	0.71	207, 0.26 (0.22-0.31)	0.23	1.08	112, 0.55 (0.46-0.66)	0.58	1.58	<.001
$20,000-<$50,000	805, 0.32 (0.30-0.35)	0.28	1.79	693, 0.31 (0.29-0.34)	0.27	1.53	444, 0.23 (0.21-0.25)	0.22	0.91	274, 0.26 (0.22-0.30)	0.24	0.89	261, 0.44 (0.38-0.51)	0.45	1.48	<.001
$50,000-<$75,000	220, 0.29 (0.25-0.35)	0.24	1.26	165, 0.27 (0.24-0.30)	0.24	0.98	82, 0.21 (0.19-0.24)	0.19	0.85	78, 0.26 (0.21-0.33)	0.25	1.28	94, 0.40 (0.33-0.50)	0.41	1.22	<.001
≥$75,000	703, 0.25 (0.23-0.27)	0.22	1.10	374, 0.27 (0.25-0.30)	0.24	1.19	104, 0.23 (0.20-0.27)	0.21	0.81	133, 0.22 (0.18-0.27)	0.19	0.88	364, 0.35 (0.32-0.38)	0.33	1.04	<.001
p-value [a]	<.001			0.001			0.201			0.321			<.001			
Poverty to Income Ratio																
≤ Median (1.63)	970, 0.34 (0.29-0.41)	0.28	2.19	942, 0.32 (0.30-0.36)	0.27	1.60	547, 0.23 (0.20-0.25)	0.21	0.81	372, 0.25 (0.22-0.29)	0.23	0.99	241, 0.49 (0.43-0.56)	0.50	1.75	<.001
> Median (1.63)	1299, 0.27 (0.26-0.29)	0.24	1.24	843, 0.29 (0.27-0.31)	0.25	1.30	291, 0.23 (0.21-0.24)	0.21	0.85	312, 0.25 (0.22-0.28)	0.23	1.06	584, 0.38 (0.34-0.41)	0.37	1.20	<.001
	0.014			0.054			0.797			0.747			<.001			
Birthplace																
U.S.	2275, 0.29 (0.27-0.31)	0.25	1.69	1790, 0.31 (0.29-0.33)	0.26	1.52	538, 0.21 (0.19-0.23)	0.18	0.89	293, 0.22 (0.19-0.26)	0.19	0.94	277, 0.24 (0.21-0.28)	0.23	0.78	<.001
Outside U.S.	99, 0.32 (0.26-0.40)	0.26	1.17	167, 0.29 (0.24-0.35)	0.28	0.83	380, 0.25 (0.23-0.27)	0.24	0.79	460, 0.28 (0.25-0.31)	0.26	1.05	668, 0.48 (0.44-0.52)	0.47	1.46	<.001
p-value [a]	0.312			0.572			0.003			0.002			<.001			
BMI																
Underweight	52, 0.37 (0.25-0.54)	0.29	2.87	42, 0.31 (0.20-0.47)	0.24	1.62	14, 0.19 (0.14-0.24)	0.18	‡	13, 0.25 (0.14-0.45)	0.19	‡	42, 0.42 (0.31-0.57)	0.45	1.38	0.004
Normal	859, 0.29 (0.25-0.33)	0.24	1.79	660, 0.28 (0.26-0.30)	0.23	1.49	321, 0.20 (0.19-0.21)	0.18	0.67	254, 0.24 (0.21-0.28)	0.22	1.11	569, 0.40 (0.36-0.45)	0.40	1.40	<.001
Overweight	712, 0.28 (0.26-0.31)	0.25	1.50	474, 0.34 (0.30-0.37)	0.29	1.49	248, 0.25 (0.22-0.28)	0.24	1.01	233, 0.26 (0.23-0.31)	0.25	1.05	228, 0.43 (0.37-0.49)	0.41	1.28	<.001
Obese	719, 0.30 (0.27-0.33)	0.26	1.59	757, 0.31 (0.29-0.33)	0.27	1.51	324, 0.23 (0.21-0.26)	0.21	0.82	253, 0.25 (0.23-0.28)	0.23	0.92	93, 0.36 (0.31-0.43)	0.31	1.42	<.001
p-value [a]	0.270			0.011			0.009			0.776			0.424			
Smoking (cotinine level)																
1st tertile	846, 0.22 (0.21-0.24)	0.22	0.56	364, 0.24 (0.23-0.26)	0.24	0.68	371, 0.19 (0.18-0.21)	0.18	0.52	314, 0.21 (0.20-0.23)	0.20	0.65	339, 0.33 (0.31-0.36)	0.32	1.06	<.001
2nd tertile	601, 0.22 (0.19-0.24)	0.20	0.65	621, 0.22 (0.21-0.24)	0.22	0.59	343, 0.20 (0.19-0.22)	0.20	0.51	250, 0.22 (0.20-0.24)	0.21	0.65	427, 0.41 (0.36-0.45)	0.39	1.19	<.001
3rd tertile	873, 0.53 (0.47-0.60)	0.56	2.88	896, 0.42 (0.39-0.46)	0.39	1.83	183, 0.37 (0.32-0.41)	0.34	1.61	174, 0.40 (0.36-0.45)	0.38	1.72	160, 0.62 (0.53-0.73)	0.62	2.07	<.001
p-value [a]	<.001			<.001			<.001			<.001			<.001			
Fish Consumption (eaten during past 30 days)																
Yes	1490, 0.30 (0.29-0.32)	0.26	1.64	1277, 0.32 (0.30-0.34)	0.28	1.50	472, 0.23 (0.21-0.26)	0.22	0.87	430, 0.25 (0.23-0.28)	0.23	0.93	605, 0.40 (0.36-0.45)	0.40	1.34	<.001
No	774, 0.27 (0.24-0.30)	0.21	1.71	543, 0.27 (0.25-0.30)	0.23	1.48	374, 0.22 (0.19-0.24)	0.20	0.88	247, 0.24 (0.20-0.30)	0.22	1.29	189, 0.31 (0.27-0.35)	0.31	1.05	<.001
p-value [a]	0.006			0.002			0.322			0.677			0.002			
Urbanization [c]																
Metro Center	0.29 (0.25-0.34)	0.26	1.56	0.32 (0.30-0.35)	0.29	1.50	0.24 (0.23-0.26)	0.23	0.79	0.27 (0.23-0.31)	0.25	1.06	0.44 (0.39-0.50)	0.44	1.42	<.001
Metro Fringe	0.30 (0.28-0.32)	0.26	1.39	0.28 (0.26-0.31)	0.25	1.39	0.25 (0.23-0.27)	0.26	0.90	0.23 (0.21-0.25)	0.19	0.81	0.33 (0.31-0.36)	0.31	1.11	<.001
Other	0.29 (0.25-0.33)	0.24	1.79	0.31 (0.28-0.35)	0.26	1.61	0.21 (0.19-0.25)	0.18	0.89	0.22 (0.18-0.28)	0.19	0.98	0.38 (0.25-0.59)	0.37	1.23	<.001
p-value [a]	0.890			0.161			0.186			0.169			0.005			
US Census Region [c]																
Northeast	0.30 (0.27-0.33)	0.26	1.35	0.33 (0.28-0.39)	0.31	1.46	0.25 (0.22-0.27)	0.26	0.70	0.27 (0.22-0.34)	0.25	1.19	0.37 (0.32-0.44)	0.36	1.40	<.001
Midwest	0.28 (0.25-0.31)	0.24	1.69	0.32 (0.28-0.38)	0.27	1.60	0.31 (0.28-0.34)	0.27	1.41	0.20 (0.14-0.28)	0.16	‡	0.32 (0.25-0.39)	0.29	0.98	<.001

	Non-Hispanic White		Non-Hispanic Black		Mexican American		Other Hispanic		Non-Hispanic Asian		p-value [b]
	N, Geometric Mean (95% CI)	Percentile 50th 95th	N, Geometric Mean (95% CI)	Percentile 50th 95th	N, Geometric Mean (95% CI)	Percentile 50th 95th	N, Geometric Mean (95% CI)	Percentile 50th 95th	N, Geometric Mean (95% CI)	Percentile 50th 95th	
South	0.32 (0.27-0.39)	0.28 1.71	0.30 (0.28-0.32)	0.26 1.50	0.25 (0.23-0.27)	0.24 0.79	0.26 (0.24-0.28)	0.24 1.09	0.43 (0.35-0.52)	0.40 1.23	<.001
West	0.27 (0.25-0.30)	0.22 1.52	0.34 (0.26-0.44)	0.33 1.38	0.21 (0.18-0.24)	0.18 0.83	0.21 (0.16-0.26)	0.18 0.73	0.43 (0.36-0.52)	0.43 1.49	<.001
p-value [a]	0.394		0.451		0.001		0.141		0.110		

† Not calculated due to high frequency of non-detected results (below limit of detection). ‡ Not calculated due to small number of samples. [a] Significance of difference in geometric mean across categories within covariate. [b] Significance of difference in geometric mean across NHANES racial and ethnic groups. [c] Raw sample counts are not provided for the restricted data. Abbreviations: NHANES - National Health and Nutrition Examination Survey, GED - General Education Development, AA - Associate of Arts, BMI - Body Mass Index, CDC - Centers for Disease Control and Prevention, NCHS - National Center for Health Statistics.

Table S2. Weighted statistics of blood lead levels (µg/dL) by NHANES racial and ethnic group

	Non-Hispanic White			Non-Hispanic Black			Mexican American			Other Hispanic			Non-Hispanic Asian			p-value [b]
	N, Geometric Mean (95% CI)	Percentile 50th	Percentile 95th	N, Geometric Mean (95% CI)	Percentile 50th	Percentile 95th	N, Geometric Mean (95% CI)	Percentile 50th	Percentile 95th	N, Geometric Mean (95% CI)	Percentile 50th	Percentile 95th	N, Geometric Mean (95% CI)	Percentile 50th	Percentile 95th	
Overall	2374, 1.00 (0.92-1.08)	0.95	3.12	1957, 0.98 (0.93-1.03)	0.89	3.69	920, 0.83 (0.76-0.91)	0.77	3.07	755, 0.88 (0.79-0.98)	0.84	2.76	945, 1.16 (1.07-1.25)	1.15	3.29	<.001
Sex																
Male	1205, 1.16 (1.06-1.27)	1.11	3.64	949, 1.16 (1.06-1.27)	1.03	4.83	479, 1.00 (0.87-1.15)	0.93	3.35	361, 1.07 (0.93-1.23)	1.05	3.42	471, 1.31 (1.19-1.44)	1.31	3.65	0.005
Female	1169, 0.86 (0.79-0.93)	0.84	2.56	1008, 0.85 (0.81-0.90)	0.80	3.12	441, 0.68 (0.63-0.74)	0.61	2.18	394, 0.74 (0.67-0.80)	0.72	1.93	474, 1.04 (0.95-1.14)	1.00	2.92	<.001
p-value [a]	<.001			<.001			<.001			<.001			<.001			
Age																
6-11 yrs.	242, 0.63 (0.55-0.71)	0.59	1.78	320, 0.90 (0.77-1.04)	0.82	2.91	222, 0.65 (0.58-0.72)	0.64	1.46	114, 0.72 (0.60-0.86)	0.70	1.74	89, 0.80 (0.65-0.99)	0.75	2.71	<.001
12-19 yrs.	251, 0.54 (0.48-0.61)	0.50	1.24	346, 0.59 (0.56-0.63)	0.58	1.31	199, 0.52 (0.47-0.59)	0.50	1.24	125, 0.56 (0.48-0.67)	0.53	1.91	155, 0.71 (0.61-0.82)	0.69	1.73	0.007
20-39 yrs.	622, 0.71 (0.65-0.78)	0.68	1.91	415, 0.71 (0.66-0.77)	0.65	2.37	213, 0.86 (0.75-1.00)	0.76	3.36	161, 0.79 (0.68-0.92)	0.77	2.08	293, 1.01 (0.91-1.11)	0.95	2.80	<.001
40-59 yrs.	571, 1.21 (1.10-1.33)	1.13	3.16	454, 1.36 (1.21-1.53)	1.25	5.79	180, 1.16 (1.05-1.27)	1.17	3.38	168, 1.11 (1.01-1.24)	1.03	2.92	252, 1.47 (1.34-1.61)	1.42	3.85	<.001
60+ yrs.	688, 1.56 (1.42-1.72)	1.52	4.03	422, 1.78 (1.66-1.91)	1.62	5.61	106, 1.20 (1.04-1.39)	1.09	3.84	187, 1.25 (1.11-1.40)	1.23	3.42	156, 1.62 (1.50-1.75)	1.56	3.81	<.001
p-value [a]	<.001			<.001			<.001			<.001			<.001			
Education																
<High school (HS)	394, 1.20 (1.06-1.35)	1.21	3.85	393, 1.35 (1.17-1.54)	1.21	6.47	506, 0.93 (0.84-1.03)	0.88	3.48	286, 1.04 (0.88-1.24)	0.98	3.19	147, 1.53 (1.37-1.70)	1.51	3.91	<.001
HS graduate/GED	488, 1.12 (0.96-1.30)	1.02	3.83	520, 1.11 (0.96-1.29)	1.02	4.38	186, 0.83 (0.68-1.02)	0.76	3.13	167, 0.84 (0.69-1.02)	0.83	2.63	127, 1.27 (1.09-1.48)	1.23	2.89	0.002
Some college/AA	786, 0.97 (0.87-1.08)	0.94	3.03	693, 0.85 (0.76-0.94)	0.79	2.72	162, 0.67 (0.60-0.75)	0.65	1.87	173, 0.78 (0.71-0.86)	0.80	1.81	206, 1.09 (0.95-1.25)	1.04	2.82	<.001
≥College graduate	687, 0.92 (0.86-0.98)	0.89	2.59	324, 0.82 (0.71-0.94)	0.76	2.51	61, 0.68 (0.58-0.79)	0.63	1.91	116, 0.80 (0.69-0.93)	0.77	2.27	456, 1.08 (0.99-1.18)	1.06	3.02	<.001
p-value [a]	<.001			<.001			<.001			<.001			<.001			
Household Income																
<\$20,000	557, 1.09 (0.88-1.33)	1.05	3.55	584, 1.09 (1.00-1.19)	0.94	5.68	240, 0.90 (0.80-1.01)	0.78	3.35	207, 0.98 (0.89-1.07)	0.95	3.16	112, 1.15 (0.98-1.36)	1.24	2.89	0.006
\$20,000-<\$50,000	805, 1.02 (0.90-1.15)	1.00	3.34	693, 0.95 (0.88-1.02)	0.88	3.10	444, 0.86 (0.75-1.00)	0.81	3.18	274, 0.90 (0.75-1.07)	0.88	2.40	261, 1.22 (1.07-1.40)	1.22	3.17	<.001
\$50,000-<\$75,000	220, 1.00 (0.90-1.10)	0.98	2.60	165, 0.86 (0.78-0.95)	0.82	2.24	82, 0.70 (0.60-0.83)	0.66	2.43	78, 0.85 (0.71-1.01)	0.82	2.70	94, 1.18 (0.98-1.41)	1.18	2.66	<.001
≥\$75,000	703, 0.94 (0.84-1.04)	0.88	2.76	374, 0.89 (0.82-0.96)	0.82	2.64	104, 0.68 (0.57-0.82)	0.65	1.82	133, 0.74 (0.61-0.90)	0.72	2.78	364, 1.11 (1.02-1.20)	1.05	3.45	<.001
p-value [a]	0.358			0.002			0.002			0.013			0.647			
Poverty to Income Ratio																
≤ Median (1.63)	970, 0.98 (0.83-1.14)	0.93	3.14	942, 0.99 (0.94-1.04)	0.87	4.04	547, 0.89 (0.76-1.03)	0.79	3.34	372, 0.94 (0.83-1.08)	0.91	2.94	241, 1.22 (1.06-1.39)	1.23	3.24	0.053
> Median (1.63)	1299, 0.99 (0.93-1.05)	0.96	2.95	843, 0.94 (0.88-1.00)	0.89	2.93	291, 0.75 (0.68-0.82)	0.69	2.13	312, 0.82 (0.71-0.94)	0.81	2.74	584, 1.13 (1.04-1.23)	1.12	3.30	<.001
	0.860			0.103			0.030			0.022			0.343			
Birthplace																
U.S.	2275, 0.99 (0.92-1.08)	0.95	3.07	1790, 0.96 (0.91-1.02)	0.86	3.75	538, 0.65 (0.58-0.73)	0.62	1.82	293, 0.67 (0.62-0.73)	0.67	1.74	277, 0.79 (0.72-0.86)	0.77	2.18	<.001
Outside U.S.	99, 1.03 (0.77-1.40)	0.88	3.95	167, 1.19 (1.09-1.29)	1.10	2.86	380, 1.10 (0.97-1.26)	1.00	4.01	460, 1.03 (0.91-1.17)	0.98	3.02	668, 1.32 (1.24-1.41)	1.29	3.56	0.002
p-value [a]	0.747			0.001			<.001			<.001			<.001			
BMI																
Underweight	52, 1.02 (0.80-1.30)	0.89	5.53	42, 1.20 (0.88-1.63)	0.80	5.76	14, 0.67 (0.42-1.06)	0.62	‡	13, 0.83 (0.58-1.18)	0.81	‡	42, 1.08 (0.87-1.35)	1.00	2.24	0.131
Normal	859, 0.94 (0.83-1.05)	0.90	2.97	660, 0.92 (0.85-1.00)	0.82	3.35	321, 0.75 (0.70-0.80)	0.66	2.73	254, 0.84 (0.74-0.94)	0.81	2.37	569, 1.14 (1.04-1.25)	1.11	3.30	<.001
Overweight	712, 1.06 (0.97-1.16)	1.08	3.06	474, 1.11 (1.02-1.21)	1.01	4.99	248, 0.89 (0.80-0.99)	0.87	3.26	233, 0.94 (0.81-1.11)	0.92	3.14	228, 1.26 (1.14-1.38)	1.27	3.56	<.001
Obese	719, 0.99 (0.89-1.10)	0.94	3.13	757, 0.95 (0.89-1.01)	0.89	3.30	324, 0.86 (0.72-1.04)	0.77	4.13	253, 0.87 (0.74-1.01)	0.85	2.76	93, 1.12 (0.95-1.33)	1.18	2.91	0.192
p-value [a]	0.121			<.001			0.026			0.456			0.262			
Smoking (cotinine level)																
1st tertile	846, 0.89 (0.82-0.96)	0.87	2.75	364, 0.84 (0.72-0.97)	0.83	2.41	371, 0.69 (0.63-0.74)	0.65	1.96	314, 0.73 (0.67-0.80)	0.73	1.97	339, 0.94 (0.85-1.05)	0.93	2.58	<.001
2nd tertile	601, 0.95 (0.87-1.03)	0.94	2.72	621, 0.87 (0.81-0.94)	0.81	2.79	343, 0.91 (0.83-1.01)	0.84	3.58	250, 0.90 (0.76-1.08)	0.85	2.75	427, 1.29 (1.21-1.38)	1.27	3.51	<.001
3rd tertile	873, 1.18 (1.04-1.34)	1.13	4.11	896, 1.12 (1.02-1.23)	0.99	5.04	183, 1.01 (0.85-1.20)	0.89	3.62	174, 1.13 (0.97-1.32)	1.07	3.78	160, 1.39 (1.22-1.59)	1.37	3.61	0.014
p-value [a]	<.001			0.002			<.001			<.001			<.001			
Fish Consumption (eaten during past 30 days)																
Yes	1490, 1.08 (1.00-1.17)	1.05	3.13	1277, 1.04 (1.00-1.09)	0.95	3.98	472, 0.86 (0.79-0.93)	0.76	3.09	430, 0.91 (0.83-1.00)	0.89	2.77	605, 1.15 (1.05-1.25)	1.13	3.02	<.001
No	774, 0.83 (0.73-0.95)	0.75	3.08	543, 0.84 (0.76-0.92)	0.73	3.04	374, 0.81 (0.70-0.93)	0.77	3.23	247, 0.83 (0.71-0.96)	0.78	2.81	189, 1.04 (0.93-1.16)	1.05	3.34	0.005
p-value [a]	<.001			<.001			0.341			0.097			0.041			
Urbanization [c]																
Metro Center	0.89 (0.82-0.97)	0.87	2.42	0.93 (0.85-1.02)	0.86	3.02	0.81 (0.69-0.94)	0.71	3.35	0.87 (0.73-1.04)	0.85	2.48	1.20 (1.08-1.35)	1.22	3.28	<.001
Metro Fringe	1.10 (0.86-1.40)	1.05	3.20	1.05 (1.00-1.10)	0.98	3.63	1.29 (0.89-1.87)	1.07	9.61	0.95 (0.85-1.06)	0.86	3.01	1.06 (0.91-1.23)	1.02	3.65	<.001
Other	1.00 (0.88-1.13)	0.95	3.30	1.00 (0.96-1.04)	0.83	5.94	0.82 (0.71-0.96)	0.78	2.27	0.82 (0.74-0.90)	0.71	2.38	1.14 (1.02-1.27)	1.08	3.03	<.001
p-value [a]	0.139			0.104			0.064			0.134			0.398			
US Census Region [c]																
Northeast	1.28 (1.05-1.57)	1.24	5.18	1.06 (1.00-1.12)	0.98	3.18	1.28 (0.84-1.96)	1.11	9.56	0.95 (0.86-1.05)	0.91	2.94	1.23 (1.06-1.42)	1.17	3.80	<.001
Midwest	0.96 (0.81-1.13)	0.93	3.07	1.06 (0.96-1.17)	1.00	3.77	0.92 (0.74-1.14)	0.86	3.24	0.95 (0.72-1.26)	0.71	‡	0.96 (0.76-1.20)	0.95	2.87	0.129

	Non-Hispanic White		Non-Hispanic Black		Mexican American		Other Hispanic		Non-Hispanic Asian		p-value [b]
	N, Geometric Mean (95% CI)	Percentile 50th 95th	N, Geometric Mean (95% CI)	Percentile 50th 95th	N, Geometric Mean (95% CI)	Percentile 50th 95th	N, Geometric Mean (95% CI)	Percentile 50th 95th	N, Geometric Mean (95% CI)	Percentile 50th 95th	
South	1.00 (0.89-1.12)	0.97 3.07	0.96 (0.89-1.04)	0.86 3.87	0.93 (0.90-0.97)	0.84 3.69	0.89 (0.71-1.12)	0.85 2.73	1.27 (1.14-1.42)	1.28 3.62	<.001
West	0.90 (0.84-0.97)	0.85 2.76	0.91 (0.82-1.00)	0.80 3.31	0.73 (0.65-0.81)	0.68 2.13	0.70 (0.58-0.83)	0.64 1.89	1.09 (0.96-1.24)	1.04 2.78	<.001
p-value [a]	0.029		0.035		0.002		0.034		0.037		

‡ Not calculated due to small number of samples. [a] Significance of difference in geometric mean across categories within covariate. [b] Significance of difference in geometric mean across NHANES racial and ethnic groups. [c] Raw sample counts are not provided for the restricted data. Abbreviations: NHANES - National Health and Nutrition Examination Survey, GED - General Education Development, AA - Associate of Arts, BMI - Body Mass Index, CDC - Centers for Disease Control and Prevention, NCHS - National Center for Health Statistics.

Table S3. Weighted statistics of blood mercury (total) levels (µg/L) by NHANES racial and ethnic group

	Non-Hispanic White			Non-Hispanic Black			Mexican American			Other Hispanic			Non-Hispanic Asian			p-value [b]
	N, Geometric Mean (95% CI)	Percentile 50th	95th	N, Geometric Mean (95% CI)	Percentile 50th	95th	N, Geometric Mean (95% CI)	Percentile 50th	95th	N, Geometric Mean (95% CI)	Percentile 50th	95th	N, Geometric Mean (95% CI)	Percentile 50th	95th	
Overall	2374, 0.71 (0.61-0.84)	0.65	4.29	1957, 0.71 (0.57-0.89)	0.65	3.98	920, 0.51 (0.45-0.58)	0.50	1.92	755, 0.91 (0.81-1.02)	0.88	4.16	945, 1.93 (1.65-2.27)	2.36	10.54	<.001
Sex																
Male	1205, 0.74 (0.62-0.88)	0.68	4.77	949, 0.71 (0.60-0.84)	0.63	4.51	479, 0.52 (0.44-0.61)	0.49	2.11	361, 0.90 (0.77-1.06)	0.87	4.79	471, 1.96 (1.67-2.30)	2.38	9.93	<.001
Female	1169, 0.69 (0.59-0.81)	0.63	3.90	1008, 0.72 (0.55-0.95)	0.68	3.47	441, 0.51 (0.46-0.56)	0.51	1.74	394, 0.91 (0.80-1.04)	0.88	3.69	474, 1.91 (1.60-2.28)	2.35	10.53	<.001
p-value [a]	0.041			0.783			0.728			0.895			0.617			
Age																
6-11 yrs.	242, 0.28 (0.22-0.36)	0.28	1.09	320, 0.39 (0.33-0.46)	0.38	1.46	222, 0.32 (0.27-0.38)	0.31	1.01	114, 0.44 (0.36-0.54)	0.41	1.74	89, 0.80 (0.65-0.99)	0.81	3.53	<.001
12-19 yrs.	251, 0.36 (0.29-0.45)	0.32	1.74	346, 0.47 (0.40-0.55)	0.47	1.88	199, 0.38 (0.33-0.45)	0.35	1.53	125, 0.54 (0.48-0.61)	0.56	2.23	155, 1.09 (0.89-1.33)	1.19	5.04	<.001
20-39 yrs.	622, 0.65 (0.50-0.84)	0.60	4.07	415, 0.71 (0.55-0.92)	0.65	3.78	213, 0.56 (0.49-0.65)	0.55	1.59	161, 0.91 (0.78-1.07)	0.87	3.78	293, 1.60 (1.33-1.93)	1.95	7.51	<.001
40-59 yrs.	571, 0.88 (0.74-1.04)	0.82	3.85	454, 0.97 (0.76-1.24)	0.89	5.51	180, 0.75 (0.66-0.86)	0.71	2.69	168, 1.23 (1.08-1.41)	1.16	4.65	252, 2.92 (2.35-3.63)	3.35	17.06	<.001
60+ yrs.	688, 1.00 (0.75-1.31)	0.91	7.85	422, 0.91 (0.74-1.12)	0.89	4.41	106, 0.43 (0.36-0.52)	0.39	1.82	187, 1.22 (0.95-1.57)	1.24	5.69	156, 2.74 (2.14-3.50)	3.30	10.89	<.001
p-value [a]	<.001			<.001			<.001			<.001			<.001			
Education																
<High school (HS)	394, 0.45 (0.41-0.51)	0.42	2.49	393, 0.72 (0.60-0.87)	0.70	3.53	506, 0.49 (0.43-0.56)	0.47	1.84	286, 0.89 (0.77-1.03)	0.84	3.92	147, 2.12 (1.45-3.09)	2.76	12.05	<.001
HS graduate/GED	488, 0.59 (0.49-0.71)	0.55	3.13	520, 0.67 (0.51-0.86)	0.60	3.17	186, 0.50 (0.40-0.62)	0.48	1.70	167, 0.90 (0.76-1.07)	0.86	3.72	127, 3.09 (2.24-4.27)	3.26	13.62	<.001
Some college/AA	786, 0.65 (0.57-0.75)	0.62	3.47	693, 0.66 (0.53-0.83)	0.61	3.80	162, 0.51 (0.45-0.59)	0.52	1.87	173, 0.86 (0.72-1.03)	0.83	3.68	206, 2.00 (1.54-2.60)	2.42	9.19	<.001
≥College graduate	687, 1.01 (0.80-1.29)	1.00	6.21	324, 0.95 (0.72-1.25)	0.89	4.90	61, 0.76 (0.57-1.00)	0.72	2.83	116, 1.08 (0.80-1.45)	1.13	6.52	456, 1.67 (1.36-2.05)	1.98	9.15	<.001
p-value [a]	<.001			<.001			0.023			0.502			0.012			
Household Income																
<$20,000	557, 0.51 (0.43-0.59)	0.52	2.17	584, 0.62 (0.51-0.76)	0.56	2.73	240, 0.54 (0.46-0.64)	0.49	2.10	207, 0.90 (0.70-1.16)	0.90	3.74	112, 2.63 (1.93-3.59)	2.67	16.31	<.001
$20,000-<$50,000	805, 0.58 (0.51-0.66)	0.53	2.74	693, 0.67 (0.54-0.82)	0.64	2.87	444, 0.47 (0.40-0.54)	0.46	1.72	274, 0.81 (0.67-0.98)	0.75	3.57	261, 1.75 (1.33-2.31)	2.08	10.26	<.001
$50,000-<$75,000	220, 0.60 (0.52-0.68)	0.62	2.65	165, 0.82 (0.54-1.24)	0.68	5.15	82, 0.48 (0.39-0.60)	0.55	1.44	78, 1.05 (0.81-1.34)	1.11	4.76	94, 1.60 (1.15-2.22)	1.96	7.53	<.001
≥$75,000	703, 0.98 (0.79-1.22)	0.99	6.20	374, 0.88 (0.71-1.10)	0.78	5.25	104, 0.64 (0.45-0.92)	0.66	2.62	133, 0.90 (0.74-1.09)	0.81	4.82	364, 1.86 (1.57-2.20)	2.22	9.14	<.001
p-value [a]	<.001			0.008			0.121			0.377			0.084			
Poverty to Income Ratio																
≤ Median (1.63)	970, 0.49 (0.43-0.55)	0.46	2.20	942, 0.61 (0.50-0.75)	0.58	2.71	547, 0.48 (0.42-0.56)	0.47	1.87	372, 0.81 (0.65-1.01)	0.77	3.52	241, 2.37 (1.74-3.23)	2.70	13.39	<.001
> Median (1.63)	1299, 0.82 (0.69-0.97)	0.76	4.95	843, 0.83 (0.67-1.03)	0.75	4.89	291, 0.54 (0.47-0.61)	0.55	1.91	312, 0.98 (0.86-1.11)	0.93	4.71	584, 1.72 (1.48-1.99)	2.05	8.97	<.001
	<.001			<.0001			0.060			0.107			0.040			
Birthplace																
U.S.	2275, 0.71 (0.60-0.84)	0.64	4.28	1790, 0.66 (0.54-0.79)	0.62	2.90	538, 0.42 (0.37-0.48)	0.42	1.49	293, 0.62 (0.52-0.74)	0.55	2.90	277, 1.52 (1.29-1.80)	1.66	6.71	<.001
Outside U.S.	99, 0.93 (0.68-1.25)	0.94	4.39	167, 1.81 (1.13-2.88)	1.87	8.82	380, 0.65 (0.57-0.73)	0.60	2.30	460, 1.13 (0.99-1.29)	1.05	4.53	668, 2.09 (1.74-2.51)	2.56	11.03	<.001
p-value [a]	0.102			<.001			<.0001			<.001			<.001			
BMI																
Underweight	52, 0.47 (0.33-0.67)	0.41	2.26	42, 0.55 (0.37-0.80)	0.57	2.07	14, 0.30 (0.16-0.56)	0.29	‡	13, 0.57 (0.38-0.86)	0.47	‡	42, 1.43 (0.86-2.39)	1.79	6.62	0.002
Normal	859, 0.69 (0.54-0.88)	0.60	4.38	660, 0.60 (0.51-0.70)	0.55	3.04	321, 0.47 (0.40-0.56)	0.45	2.03	254, 0.79 (0.68-0.92)	0.72	4.00	569, 2.01 (1.70-2.37)	2.44	10.56	<.001
Overweight	712, 0.86 (0.73-1.01)	0.80	4.97	474, 0.79 (0.59-1.05)	0.72	4.67	248, 0.55 (0.48-0.63)	0.53	1.98	233, 1.12 (1.00-1.25)	1.08	4.93	228, 2.13 (1.67-2.73)	2.48	11.41	<.001
Obese	719, 0.63 (0.56-0.71)	0.60	3.23	757, 0.77 (0.60-0.99)	0.71	4.08	324, 0.53 (0.48-0.58)	0.53	1.70	253, 0.86 (0.71-1.05)	0.84	3.79	93, 1.46 (1.09-1.94)	1.99	7.54	<.001
p-value [a]	<.001			0.009			0.018			0.006			0.065			
Smoking (cotinine level)																
1st tertile	846, 0.84 (0.67-1.07)	0.80	5.12	364, 0.74 (0.57-0.98)	0.63	4.87	371, 0.50 (0.44-0.57)	0.48	1.96	314, 0.98 (0.86-1.13)	0.92	4.53	339, 2.07 (1.83-2.35)	2.28	8.82	<.001
2nd tertile	601, 0.70 (0.57-0.86)	0.62	4.67	621, 0.77 (0.56-1.05)	0.69	4.72	343, 0.56 (0.47-0.66)	0.53	1.98	250, 0.82 (0.68-0.99)	0.74	3.91	427, 1.76 (1.34-2.32)	2.37	10.54	<.001
3rd tertile	873, 0.59 (0.51-0.69)	0.54	2.76	896, 0.68 (0.57-0.81)	0.65	2.77	183, 0.47 (0.40-0.56)	0.48	1.55	174, 0.93 (0.80-1.07)	0.94	3.74	160, 2.08 (1.57-2.75)	2.56	10.69	<.001
p-value [a]	0.010			0.503			0.138			0.145			0.443			
Fish Consumption (eaten during past 30 days)																
Yes	1490, 1.01 (0.84-1.22)	0.96	5.13	1277, 0.86 (0.69-1.08)	0.77	4.55	472, 0.64 (0.57-0.72)	0.64	2.32	430, 1.19 (1.06-1.35)	1.12	4.53	605, 2.61 (2.33-2.92)	2.64	11.05	<.001
No	774, 0.33 (0.29-0.37)	0.31	1.30	543, 0.43 (0.35-0.52)	0.41	1.52	374, 0.37 (0.32-0.43)	0.37	1.38	247, 0.53 (0.46-0.62)	0.50	1.98	189, 0.52 (0.43-0.64)	0.40	3.90	<.001
p-value [a]	<.001			<.0001			<.001			<.001			<.0001			
Urbanization [c]																
Metro Center	0.94 (0.73-1.21)	0.93	5.61	0.70 (0.54-0.90)	0.67	3.02	0.55 (0.48-0.63)	0.53	2.12	0.95 (0.83-1.10)	0.91	4.12	2.08 (1.57-2.77)	2.59	10.57	<.001
Metro Fringe	0.99 (0.62-1.58)	0.96	7.61	0.94 (0.68-1.29)	0.82	5.32	0.56 (0.33-0.95)	0.42	2.68	1.02 (0.82-1.26)	1.00	4.56	2.00 (1.46-2.73)	2.22	11.44	<.001
Other	0.53 (0.49-0.57)	0.51	2.56	0.55 (0.49-0.61)	0.50	2.59	0.47 (0.35-0.64)	0.48	1.48	0.51 (0.37-0.69)	0.47	1.54	1.14 (0.92-1.42)	1.27	4.18	<.001
p-value [a]	<.001			0.012			0.589			0.002			0.004			
US Census Region [c]																
Northeast	1.42 (1.07-1.87)	1.49	10.95	1.45 (1.22-1.71)	1.46	6.33	0.64 (0.39-1.05)	0.48	2.81	1.13 (1.03-1.23)	1.16	4.78	2.64 (2.04-3.42)	2.86	13.26	<.001
Midwest	0.54 (0.44-0.66)	0.53	2.61	0.57 (0.53-0.60)	0.53	2.58	0.56 (0.39-0.80)	0.57	1.43	0.56 (0.43-0.74)	0.55	‡	1.21 (0.97-1.51)	1.19	6.78	<.001

	Non-Hispanic White			Non-Hispanic Black			Mexican American			Other Hispanic			Non-Hispanic Asian			p-value [b]
	N, Geometric Mean (95% CI)	Percentile 50th	95th	N, Geometric Mean (95% CI)	Percentile 50th	95th	N, Geometric Mean (95% CI)	Percentile 50th	95th	N, Geometric Mean (95% CI)	Percentile 50th	95th	N, Geometric Mean (95% CI)	Percentile 50th	95th	
South	0.64 (0.53-0.77)	0.60	3.35	0.68 (0.50-0.94)	0.63	3.52	0.47 (0.36-0.60)	0.46	1.93	0.86 (0.68-1.10)	0.83	4.12	1.32 (1.05-1.66)	1.67	8.11	<.001
West	0.77 (0.62-0.96)	0.71	3.97	0.56 (0.42-0.76)	0.50	2.28	0.54 (0.43-0.67)	0.52	1.85	0.68 (0.51-0.91)	0.67	3.15	2.27 (1.58-3.27)	2.65	10.57	<.001
p-value [a]	<.001			<.001			0.636			<.001			0.001			

‡ Not calculated due to small number of samples. [a] Significance of difference in geometric mean across categories within covariate. [b] Significance of difference in geometric mean across NHANES racial and ethnic groups. [c] Raw sample counts are not provided for the restricted data. Abbreviations: NHANES - National Health and Nutrition Examination Survey, GED - General Education Development, AA - Associate of Arts, BMI - Body Mass Index, CDC - Centers for Disease Control and Prevention, NCHS - National Center for Health Statistics.

Table S4. Weighted statistics of urinary arsenic (total) levels (µg/g-creatinine) by NHANES racial and ethnic group

	Non-Hispanic White			Non-Hispanic Black			Mexican American			Other Hispanic			Non-Hispanic Asian			p-value [b]
	N, Geometric Mean (95% CI)	Percentile 50th	Percentile 95th	N, Geometric Mean (95% CI)	Percentile 50th	Percentile 95th	N, Geometric Mean (95% CI)	Percentile 50th	Percentile 95th	N, Geometric Mean (95% CI)	Percentile 50th	Percentile 95th	N, Geometric Mean (95% CI)	Percentile 50th	Percentile 95th	
Overall	818, 7.13 (6.05-8.39)	5.72	46.1	669, 7.24 (5.53-9.48)	5.83	54.7	317, 8.00 (6.87-9.32)	6.90	40.1	256, 9.25 (8.17-10.49)	8.72	32.9	353, 22.3 (19.1-26.1)	20.1	158.8	<.001
Sex																
Male	409, 6.58 (5.27-8.20)	5.40	44.0	342, 6.68 (5.02-8.88)	5.31	53.6	171, 7.71 (6.29-9.43)	6.39	34.9	112, 8.65 (7.53-9.94)	8.49	30.1	184, 19.2 (16.4-22.5)	17.0	133.9	<.001
Female	409, 7.69 (6.57-9.03)	6.01	46.2	327, 7.76 (5.75-10.46)	6.22	54.8	146, 8.39 (6.38-11.02)	7.09	40.8	144, 9.75 (8.21-11.59)	8.87	33.9	169, 25.6 (21.4-30.6)	23.8	162.4	<.001
p-value [a]	0.117			0.194			0.629			0.245			<.001			
Age																
6-11 yrs.	105, 8.30 (6.30-10.91)	5.82	99.6	106, 7.67 (5.95-9.89)	6.22	31.3	92, 9.04 (7.45-10.97)	7.61	43.5	43, 9.38 (7.75-11.34)	10.60	21.5	31, 18.5 (13.5-25.4)	15.6	73.3	<.001
12-19 yrs.	91, 5.62 (3.85-8.23)	4.25	78.8	115, 4.61 (3.95-5.40)	4.06	18.7	72, 6.31 (4.89-8.15)	5.27	29.7	36, 6.97 (6.09-7.99)	6.37	20.7	56, 10.0 (8.2-12.2)	8.4	39.2	<.001
20-39 yrs.	215, 6.19 (4.80-7.99)	5.12	37.6	150, 7.11 (5.04-10.05)	5.54	72.2	67, 8.47 (6.85-10.50)	7.16	48.6	59, 9.08 (7.73-10.66)	8.76	32.6	106, 19.9 (16.3-24.4)	16.7	113.7	<.001
40-59 yrs.	193, 6.73 (5.58-8.12)	5.89	33.4	147, 8.39 (6.33-11.10)	7.04	56.4	49, 8.04 (6.61-9.77)	7.01	23.6	55, 8.73 (7.33-10.43)	7.43	36.0	97, 28.1 (20.8-37.8)	24.3	165.0	<.001
60+ yrs.	214, 9.46 (6.88-13.01)	7.84	66.5	151, 8.53 (6.22-11.68)	8.22	55.9	37, 9.16 (7.33-11.45)	7.81	49.0	63, 13.61 (9.30-19.95)	12.40	37.2	63, 35.4 (26.3-47.7)	37.2	165.7	<.001
p-value [a]	0.026			<.001			0.067			0.031			<.001			
Education																
<High school (HS)	144, 6.41 (4.84-8.49)	4.99	68.0	142, 7.76 (5.53-10.89)	5.95	59.9	172, 8.09 (7.05-9.30)	6.90	37.9	95, 10.00 (8.33-11.99)	8.79	35.4	63, 28.3 (17.8-45.0)	32.2	162.7	<.001
HS graduate/GED	154, 6.61 (4.86-8.97)	5.16	39.1	176, 6.92 (5.12-9.32)	5.65	56.0	71, 7.55 (5.79-9.86)	6.30	40.0	48, 8.39 (6.99-10.09)	8.01	33.9	53, 21.8 (17.9-26.6)	22.9	114.0	<.001
Some college/AA	266, 6.24 (5.04-7.73)	5.20	32.6	233, 6.70 (4.72-9.52)	5.36	44.2	49, 7.10 (5.63-8.95)	7.47	26.0	59, 9.02 (6.96-11.65)	8.80	27.7	81, 23.9 (18.1-31.7)	22.1	165.6	<.001
≥College graduate	246, 8.67 (7.17-10.50)	6.64	49.3	106, 8.53 (6.50-11.19)	7.41	55.3	24, 10.76 (6.57-17.62)	6.68	‡	47, 9.38 (7.42-11.82)	9.13	32.9	154, 19.9 (16.2-24.5)	16.3	134.1	<.001
p-value [a]	0.026			0.274			0.084			0.500			0.326			
Household Income																
<$20,000	191, 6.17 (4.96-7.65)	5.17	35.7	199, 6.38 (4.71-8.64)	5.55	29.7	81, 7.11 (5.89-8.60)	6.37	23.6	73, 9.86 (7.82-12.43)	8.82	43.8	51, 33.1 (22.9-48.0)	32.5	165.7	<.001
$20,000-<$50,000	283, 5.92 (4.80-7.30)	4.95	32.6	228, 6.82 (5.11-9.12)	5.66	56.1	152, 8.85 (7.49-10.45)	7.52	40.8	92, 8.77 (7.35-10.46)	8.05	26.0	93, 20.6 (16.5-25.6)	20.2	93.2	<.001
$50,000-<$75,000	68, 6.41 (5.12-8.05)	5.02	32.0	58, 6.46 (4.70-8.87)	5.19	31.1	20, 6.19 (4.58-8.40)	5.12	27.1	27, 10.02 (7.80-12.86)	9.00	‡	27, 21.0 (14.0-31.6)	18.2	118.6	<.001
≥$75,000	238, 8.73 (6.94-10.99)	6.88	55.7	131, 9.64 (6.28-14.83)	7.23	89.6	43, 8.22 (4.94-13.66)	6.92	53.6	42, 8.05 (5.96-10.91)	7.73	24.0	133, 19.1 (15.2-24.1)	16.7	122.0	<.001
p-value [a]	0.041			0.142			0.031			0.489			0.049			
Poverty to Income Ratio																
≤ Median (1.63)	331, 5.57 (4.92-6.31)	4.95	32.5	314, 6.47 (4.70-8.90)	5.55	31.1	180, 8.02 (6.72-9.56)	7.21	35.4	130, 9.14 (8.03-10.38)	8.45	33.7	93, 29.0 (21.8-38.8)	32.2	140.8	<.001
> Median (1.63)	441, 7.62 (6.45-9.02)	6.16	48.7	292, 8.09 (6.19-10.59)	6.15	66.4	100, 7.43 (5.92-9.30)	6.33	41.3	103, 9.04 (7.81-10.45)	8.96	28.5	210, 19.1 (16.4-22.4)	16.5	126.0	<.001
	<.001			0.027			0.438			0.867			0.015			
Birthplace																
U.S.	782, 7.01 (5.92-8.32)	5.62	44.1	622, 6.95 (5.25-9.19)	5.54	56.0	189, 6.95 (5.93-8.16)	6.17	40.0	96, 8.26 (6.88-9.89)	6.75	33.6	92, 15.2 (12.2-18.9)	12.8	110.0	<.001
Outside U.S.	36, 10.33 (6.26-17.05)	7.62	70.8	47, 12.19 (8.86-16.77)	10.5	39.2	127, 9.40 (7.82-11.30)	8.06	39.7	158, 9.91 (8.27-11.89)	9.04	32.5	261, 25.1 (20.9-30.1)	22.9	160.5	<.001
p-value [a]	0.161			0.017			<.001			0.111			<.001			
BMI																
Underweight	21, 11.25 (6.40-19.78)	9.97	62.6	13, 5.85 (3.75-9.10)	4.57	‡	7, 5.87 (3.70-9.33)	4.61	‡	5, 8.55 (5.98-12.24)	6.30	‡	17, 16.6 (8.7-31.7)	14.8	‡	0.025
Normal	313, 8.59 (7.16-10.32)	6.70	48.7	220, 6.21 (5.05-7.63)	5.31	53.8	124, 7.96 (6.45-9.81)	6.79	39.8	83, 8.81 (7.17-10.84)	8.78	31.0	201, 23.2 (18.6-28.9)	20.5	162.4	<.001
Overweight	235, 6.64 (5.44-8.11)	5.48	41.9	161, 7.62 (5.57-10.43)	6.82	37.9	82, 9.06 (6.88-11.91)	7.10	55.0	85, 9.77 (7.82-12.23)	8.96	32.5	98, 21.2 (18.3-24.6)	19.0	124.3	<.001
Obese	233, 5.74 (4.42-7.47)	4.94	26.3	269, 7.74 (5.40-11.10)	5.83	63.7	99, 7.40 (6.16-8.86)	6.48	31.5	82, 9.25 (7.39-11.60)	7.76	36.8	31, 16.2 (11.4-23.0)	16.6	63.4	0.003
p-value [a]	0.027			0.008			0.457			0.597			0.215			
Smoking (cotinine level)																
1st tertile	285, 8.41 (6.68-10.60)	6.40	50.9	116, 7.23 (5.41-9.64)	5.52	33.5	120, 7.91 (6.48-9.63)	6.29	52.9	104, 9.86 (8.12-11.96)	8.31	39.8	108, 23.9 (18.2-31.4)	23.8	112.5	<.001
2nd tertile	208, 6.75 (5.16-8.84)	5.42	47.6	208, 7.35 (5.31-10.14)	5.67	50.5	99, 7.91 (6.67-9.35)	7.40	27.1	79, 8.61 (7.40-10.00)	8.50	23.2	158, 19.8 (16.5-23.7)	16.8	166.9	<.001
3rd tertile	267, 5.78 (5.18-6.45)	5.02	24.8	275, 7.11 (5.46-9.27)	5.83	72.6	70, 7.82 (6.04-10.09)	6.64	35.2	55, 8.77 (7.63-10.09)	8.85	25.5	55, 24.1 (17.0-34.1)	21.4	154.0	<.001
p-value [a]	0.006			0.959			0.993			0.307			0.197			
Fish Consumption (eaten during past 30 days)																
Yes	487, 9.18 (7.51-11.23)	7.26	53.2	421, 8.67 (6.93-10.86)	7.48	60.7	158, 10.02 (8.59-11.72)	8.10	51.5	147, 10.07 (8.72-11.62)	8.91	38.4	217, 25.7 (20.8-31.8)	22.5	162.5	<.001
No	283, 4.06 (3.54-4.66)	3.92	12.5	195, 4.68 (3.53-6.20)	4.19	24.7	137, 6.24 (5.19-7.48)	5.58	17.3	83, 7.73 (6.36-9.36)	6.70	23.9	72, 8.9 (7.2-10.9)	7.9	33.9	<.001
p-value [a]	<.001			<.001			<.001			0.005			<.001			
Urbanization [c]																
Metro Center	9.02 (7.10-11.47)	7.10	46.4	7.91 (5.75-10.86)	6.24	55.3	8.45 (7.05-10.15)	6.94	40.2	9.04 (7.79-10.48)	8.67	31.6	24.9 (20.2-30.7)	23.8	166.3	<.001
Metro Fringe	8.69 (6.41-11.81)	7.08	51.6	10.40 (7.93-13.67)	8.76	76.1	5.60 (3.52-8.93)	4.76	14.5	10.35 (7.19-14.88)	9.03	34.1	19.6 (12.8-30.2)	18.8	124.7	<.001
Other	5.74 (4.68-7.04)	4.94	32.3	4.17 (3.61-4.81)	3.45	25.3	7.73 (5.80-10.28)	6.84	38.6	8.41 (7.69-9.20)	6.73	24.9	14.3 (10.3-19.8)	14.8	85.1	<.001
p-value [a]	0.017			<.001			0.294			0.387			0.041			
US Census Region [c]																
Northeast	10.77 (8.20-14.14)	8.17	70.0	11.14 (8.80-14.11)	8.73	54.9	6.17 (4.28-8.89)	5.99	‡	10.54 (8.15-13.61)	8.96	41.0	25.0 (17.8-35.2)	22.5	141.9	<.001
Midwest	5.38 (4.10-7.08)	4.63	26.3	5.77 (4.54-7.33)	4.78	27.7	4.33 (2.60-7.19)	4.50	8.0	5.65 (3.36-9.52)	4.89	‡	18.0 (9.5-34.0)	14.3	106.2	<.001

	Non-Hispanic White			Non-Hispanic Black			Mexican American			Other Hispanic			Non-Hispanic Asian			p-value [b]
	N, Geometric Mean (95% CI)	Percentile		N, Geometric Mean (95% CI)	Percentile		N, Geometric Mean (95% CI)	Percentile		N, Geometric Mean (95% CI)	Percentile		N, Geometric Mean (95% CI)	Percentile		
		50th	95th		50th	95th		50th	95th		50th	95th		50th	95th	
South	6.71 (5.32-8.46)	5.34	48.7	7.11 (4.67-10.83)	5.85	56.2	8.93 (7.94-10.03)	7.21	65.9	9.12 (7.79-10.69)	8.84	28.1	18.1 (13.8-23.8)	14.6	86.3	<.001
West	8.61 (7.28-10.18)	6.86	38.1	6.70 (4.27-10.49)	5.26	26.6	8.04 (6.55-9.87)	6.90	30.7	7.82 (6.59-9.26)	6.55	21.3	24.4 (18.3-32.6)	23.1	167.4	<.001
p-value [a]	0.010			0.009			0.060			0.188			0.336			

‡ Not calculated due to small number of samples. [a] Significance of difference in geometric mean across categories within covariate. [b] Significance of difference in geometric mean across NHANES racial and ethnic groups. [c] Raw sample counts are not provided for the restricted data. Abbreviations: NHANES - National Health and Nutrition Examination Survey, GED - General Education Development, AA - Associate of Arts, BMI - Body Mass Index, CDC - Centers for Disease Control and Prevention, NCHS - National Center for Health Statistics.

Table S5. Weighted statistics of urinary dimethylarsonic acid (DMA) levels (µg/g-creatinine) by NHANES racial and ethnic group

	Non-Hispanic White			Non-Hispanic Black			Mexican American			Other Hispanic			Non-Hispanic Asian			p-value [b]
	N, Geometric Mean (95% CI)	Percentile 50th	95th	N, Geometric Mean (95% CI)	Percentile 50th	95th	N, Geometric Mean (95% CI)	Percentile 50th	95th	N, Geometric Mean (95% CI)	Percentile 50th	95th	N, Geometric Mean (95% CI)	Percentile 50th	95th	
Overall	824, 3.68 (3.44-3.93)	3.47	11.52	672, 3.16 (2.67-3.73)	3.04	10.94	317, 4.12 (3.84-4.43)	4.09	10.41	257, 5.02 (4.50-5.61)	5.05	13.96	356, 9.89 (8.58-11.4)	9.69	44.0	<.001
Sex																
Male	410, 3.30 (2.99-3.62)	3.17	9.43	342, 2.90 (2.37-3.54)	2.82	10.11	171, 4.13 (3.48-4.91)	4.06	13.48	113, 4.62 (3.98-5.37)	4.57	10.91	184, 8.47 (7.42-9.7)	8.67	34.9	<.001
Female	414, 4.09 (3.81-4.40)	3.90	12.32	330, 3.40 (2.89-3.99)	3.19	12.33	146, 4.11 (3.69-4.58)	4.20	9.25	144, 5.36 (4.69-6.13)	5.14	14.85	172, 11.38 (9.62-13.4)	10.77	54.8	<.001
p-value [a]	<.001			0.030			0.968			0.095			<.001			
Age																
6-11 yrs.	105, 4.52 (4.17-4.89)	4.13	9.81	107, 3.95 (3.43-4.57)	3.59	11.15	92, 5.35 (4.77-5.98)	5.07	13.40	44, 5.98 (5.18-6.91)	6.02	12.49	32, 11.19 (8.35-15.0)	10.32	34.1	<.001
12-19 yrs.	91, 3.13 (2.46-3.97)	3.02	12.26	116, 2.28 (2.00-2.59)	2.09	5.58	72, 3.44 (2.93-4.03)	3.18	7.68	36, 3.94 (3.07-5.04)	4.01	10.68	57, 5.64 (4.88-6.5)	5.85	17.3	<.001
20-39 yrs.	217, 3.35 (2.90-3.86)	3.31	10.61	150, 3.11 (2.49-3.88)	3.01	11.37	67, 4.15 (3.71-4.65)	4.09	11.72	59, 4.78 (4.15-5.49)	4.89	10.55	106, 8.47 (7.21-10.0)	8.22	36.1	<.001
40-59 yrs.	193, 3.72 (3.29-4.19)	3.40	10.65	148, 3.30 (2.66-4.10)	2.98	12.28	49, 3.85 (3.11-4.79)	3.90	9.96	55, 4.72 (4.13-5.40)	4.49	12.26	98, 11.59 (8.76-15.3)	10.80	52.7	<.001
60+ yrs.	218, 4.06 (3.60-4.58)	3.80	12.65	151, 3.55 (2.96-4.27)	3.21	12.81	37, 4.74 (3.97-5.65)	4.33	11.59	63, 7.11 (5.67-8.94)	6.13	17.44	63, 14.35 (12.60-16.4)	13.87	54.2	<.001
p-value [a]	<.001			<.001			<.001			0.004			<.001			
Education																
<High school (HS)	146, 3.32 (2.83-3.89)	3.02	9.83	143, 3.11 (2.49-3.89)	3.03	10.73	172, 4.28 (3.97-4.60)	4.23	10.47	95, 5.70 (4.73-6.87)	5.46	16.36	63, 13.55 (9.22-19.9)	12.97	66.3	<.001
HS graduate/GED	156, 3.47 (2.96-4.07)	3.28	9.41	177, 3.03 (2.54-3.61)	2.89	9.69	71, 4.10 (3.57-4.72)	4.14	9.98	49, 4.51 (3.58-5.67)	4.26	11.72	53, 10.14 (8.90-11.6)	10.92	23.4	<.001
Some college/AA	267, 3.50 (3.17-3.87)	3.29	9.98	233, 3.09 (2.46-3.88)	3.04	11.63	49, 3.68 (2.89-4.68)	3.25	7.68	59, 4.83 (4.11-5.69)	4.49	9.89	81, 9.91 (7.86-12.5)	9.49	44.4	<.001
≥College graduate	247, 4.12 (3.59-4.72)	3.91	13.30	107, 3.49 (2.96-4.12)	3.11	12.37	24, 4.26 (3.38-5.36)	3.55	‡	47, 4.80 (4.04-5.70)	5.23	10.81	157, 8.71 (7.40-10.3)	7.97	34.3	<.001
p-value [a]	0.130			0.369			0.305			0.137			0.065			
Household Income																
<$20,000	193, 3.29 (2.84-3.80)	3.17	10.03	201, 2.93 (2.37-3.62)	2.89	8.65	81, 3.85 (3.48-4.26)	3.75	9.57	73, 5.48 (4.68-6.42)	5.53	14.35	52, 15.00 (10.21-22.0)	13.50	80.6	<.001
$20,000-<$50,000	285, 3.32 (2.92-3.78)	3.14	8.84	229, 3.16 (2.56-3.89)	3.06	10.83	152, 4.57 (4.24-4.93)	4.27	14.32	93, 4.99 (4.20-5.91)	4.75	15.11	93, 9.71 (7.96-11.8)	9.82	36.4	<.001
$50,000-<$75,000	70, 3.30 (2.92-3.73)	3.27	7.31	58, 2.94 (2.40-3.60)	2.79	9.26	20, 3.46 (2.70-4.42)	3.05	8.35	27, 6.11 (5.14-7.27)	6.57	10.39	27, 8.91 (6.30-12.6)	9.51	32.8	<.001
≥$75,000	238, 4.24 (3.68-4.87)	4.03	12.95	131, 3.52 (2.91-4.27)	3.11	14.49	43, 3.81 (3.22-4.49)	3.74	8.08	42, 3.78 (3.18-4.51)	3.78	10.64	135, 8.69 (7.32-10.3)	8.02	36.0	<.001
p-value [a]	0.035			0.181			0.014			<.001			0.073			
Poverty to Income Ratio																
≤ Median (1.63)	333, 3.22 (3.04-3.42)	3.08	9.07	317, 2.99 (2.38-3.76)	2.87	9.06	180, 4.20 (3.89-4.53)	4.21	9.92	130, 5.09 (4.43-5.87)	4.79	15.03	94, 13.21 (10.04-17.3)	12.98	59.5	<.001
> Median (1.63)	445, 3.80 (3.49-4.15)	3.52	11.56	292, 3.32 (2.92-3.78)	3.11	13.46	100, 3.86 (3.53-4.23)	3.64	9.68	104, 4.91 (4.41-5.47)	5.25	10.63	212, 8.75 (7.63-10.0)	8.31	35.1	<.001
	0.002			0.090			0.198			0.579			0.008			
Birthplace																
U.S.	787, 3.65 (3.40-3.91)	3.42	11.28	624, 2.99 (2.53-3.53)	2.88	10.55	189, 3.80 (3.41-4.23)	3.85	8.97	97, 4.15 (3.71-4.65)	4.27	10.59	94, 7.40 (6.41-8.5)	6.81	31.6	<.001
Outside U.S.	37, 4.58 (3.30-6.36)	4.78	11.85	48, 6.24 (4.94-7.90)	5.96	14.10	127, 4.51 (4.18-4.86)	4.20	14.10	158, 5.61 (4.76-6.61)	5.70	15.04	262, 10.81 (9.04-12.9)	10.20	52.6	<.001
p-value [a]	0.190			<.001			0.014			0.003			<.001			
BMI																
Underweight	21, 4.60 (3.25-6.53)	4.38	12.96	13, 2.44 (1.57-3.81)	1.87	‡	7, 3.42 (2.17-5.40)	3.52	‡	5, 4.97 (3.14-7.83)	4.91	‡	17, 8.65 (5.43-13.8)	7.09	‡	0.003
Normal	317, 4.07 (3.62-4.58)	3.97	13.72	221, 3.16 (2.70-3.71)	3.05	8.87	124, 4.17 (3.72-4.67)	3.87	13.14	83, 4.66 (3.91-5.55)	4.71	11.68	202, 10.00 (8.33-12.0)	9.60	45.2	<.001
Overweight	236, 3.47 (3.17-3.79)	3.27	9.93	161, 3.09 (2.50-3.82)	3.04	11.65	82, 4.42 (3.93-4.97)	4.31	9.74	85, 5.46 (4.45-6.70)	5.52	13.90	100, 9.62 (8.66-10.7)	10.33	25.1	<.001
Obese	233, 3.33 (2.92-3.80)	3.20	9.57	271, 3.20 (2.64-3.88)	3.06	12.59	99, 3.79 (3.38-4.25)	3.78	9.16	83, 5.01 (4.12-6.11)	4.95	15.17	31, 8.30 (6.05-11.4)	7.36	35.2	<.001
p-value [a]	0.096			0.188			0.359			0.421			0.611			
Smoking (cotinine level)																
1st tertile	288, 4.07 (3.59-4.63)	3.72	13.00	116, 3.54 (3.01-4.17)	3.29	10.20	120, 4.38 (3.77-5.08)	4.27	9.53	104, 5.19 (4.47-6.01)	5.08	14.90	110, 10.62 (8.10-13.9)	9.79	39.2	<.001
2nd tertile	209, 3.56 (3.18-3.98)	3.42	11.57	209, 3.04 (2.45-3.77)	2.85	12.31	99, 3.81 (3.43-4.24)	3.93	8.96	80, 4.84 (4.15-5.65)	4.69	12.33	159, 9.25 (7.87-10.9)	9.46	39.2	<.001
3rd tertile	268, 3.23 (3.06-3.41)	3.21	8.83	274, 3.06 (2.60-3.61)	2.93	10.72	70, 4.04 (3.43-4.74)	3.73	14.21	55, 4.80 (3.99-5.75)	4.55	11.87	55, 8.95 (7.14-11.2)	9.48	52.0	<.001
p-value [a]	<.001			0.123			0.131			0.377			0.545			
Fish Consumption (eaten during past 30 days)																
Yes	489, 4.17 (3.79-4.58)	4.03	11.97	421, 3.39 (2.93-3.92)	3.19	12.24	158, 4.40 (3.90-4.95)	4.08	14.10	147, 4.95 (4.25-5.77)	4.91	13.05	218, 10.23 (8.75-11.9)	9.74	40.0	<.001
No	286, 2.84 (2.62-3.09)	2.83	9.18	197, 2.73 (2.24-3.32)	2.70	8.52	137, 3.85 (3.38-4.37)	4.10	7.87	83, 4.86 (4.16-5.70)	4.75	12.67	74, 5.62 (5.01-6.3)	4.82	17.2	<.001
p-value [a]	<.001			<.001			0.152			0.875			<.001			
Urbanization [c]																
Metro Center	4.27 (3.73-4.89)	3.95	14.99	3.32 (2.75-4.00)	3.17	11.53	4.32 (3.83-4.86)	3.95	14.31	5.14 (4.45-5.93)	5.08	14.57	10.38 (9.06-11.9)	9.85	49.0	<.001
Metro Fringe	4.17 (3.40-5.11)	4.20	11.61	4.07 (3.45-4.81)	3.89	13.47	3.64 (2.77-4.78)	2.97	8.13	4.81 (3.53-6.55)	4.45	12.99	10.50 (7.04-15.6)	9.77	41.1	<.001
Other	3.21 (3.13-3.30)	3.13	9.39	2.18 (1.89-2.52)	2.02	7.38	3.95 (3.31-4.71)	4.22	9.33	4.75 (3.89-5.79)	4.54	8.57	6.38 (4.77-8.5)	6.45	17.1	<.001
p-value [a]	0.001			<.001			0.470			0.785			0.023			
US Census Region [c]																
Northeast	4.75 (4.47-5.05)	4.31	12.87	4.06 (3.58-4.59)	3.97	12.56	4.10 (3.44-4.89)	3.81	‡	5.42 (4.22-6.98)	5.13	16.58	11.35 (9.00-14.3)	10.84	41.1	<.001

	Non-Hispanic White			Non-Hispanic Black			Mexican American			Other Hispanic			Non-Hispanic Asian			p-value [b]
	N, Geometric Mean (95% CI)	Percentile 50th	95th	N, Geometric Mean (95% CI)	Percentile 50th	95th	N, Geometric Mean (95% CI)	Percentile 50th	95th	N, Geometric Mean (95% CI)	Percentile 50th	95th	N, Geometric Mean (95% CI)	Percentile 50th	95th	
Midwest	3.31 (2.99-3.68)	3.11	9.53	2.81 (1.96-4.01)	2.81	8.14	2.44 (1.90-3.14)	2.33	‡	3.58 (2.25-5.69)	3.76	‡	9.31 (5.71-15.2)	7.41	37.7	<.001
South	3.23 (3.03-3.44)	3.17	10.63	3.06 (2.38-3.94)	2.89	10.83	3.78 (3.46-4.12)	3.85	9.11	4.82 (4.03-5.78)	4.90	10.59	7.94 (5.99-10.6)	7.02	37.2	<.001
West	4.29 (3.92-4.68)	3.98	11.81	3.65 (2.68-4.96)	3.23	12.56	4.60 (4.24-5.00)	4.61	10.56	5.15 (4.23-6.29)	5.35	‡	10.42 (8.63-12.6)	10.19	47.5	<.001
p-value [a]	<.001			0.124			<.001			0.501			0.189			

‡ Not calculated due to small number of samples. [a] Significance of difference in geometric mean across categories within covariate. [b] Significance of difference in geometric mean across NHANES racial and ethnic groups. [c] Raw sample counts are not provided for the restricted data. Abbreviations: NHANES - National Health and Nutrition Examination Survey, GED - General Education Development, AA - Associate of Arts, BMI - Body Mass Index, CDC - Centers for Disease Control and Prevention, NCHS - National Center for Health Statistics.

Supplemental Material 2: Weighted statistics of biomarker levels by Asian subgroup

Table S6. Weighted statistics of blood cadmium levels (μg/L) by Asian subgroup

	Chinese			Asian Indian			Other Asian			p-value[b]
	GM (95% CI)	Percentile 50th	95th	GM (95% CI)	Percentile 50th	95th	GM (95% CI)	Percentile 50th	95th	
Overall	0.45 (0.40-0.51)	0.46	1.44	0.31 (0.28-0.34)	0.30	1.01	0.43 (0.38-0.49)	0.44	1.45	<.001
Sex										
Male	0.42 (0.35-0.50)	0.40	1.58	0.28 (0.24-0.33)	0.27	1.19	0.34 (0.29-0.41)	0.33	1.30	<.001
Female	0.49 (0.43-0.56)	0.54	1.31	0.34 (0.31-0.39)	0.32	0.92	0.53 (0.47-0.59)	0.55	1.48	<.001
p-value [a]	0.098			0.030			<.001			
Age										
6-11 yrs.	0.20 (0.16-0.24)	0.23	‡	0.14 (0.12-0.17)	†	0.23	0.14 (0.12-0.17)	†	0.35	0.056
12-19 yrs.	0.20 (0.16-0.27)	0.19	0.38	0.22 (0.16-0.30)	0.22	0.57	0.21 (0.18-0.25)	0.21	0.57	0.909
20-39 yrs.	0.46 (0.38-0.57)	0.41	1.39	0.29 (0.25-0.33)	0.28	0.91	0.42 (0.39-0.47)	0.39	1.43	<.001
40-59 yrs.	0.57 (0.48-0.69)	0.56	1.49	0.38 (0.32-0.44)	0.36	1.02	0.55 (0.49-0.62)	0.52	1.52	<.001
60+ yrs.	0.62 (0.52-0.74)	0.61	1.45	0.41 (0.30-0.55)	0.36	‡	0.74 (0.61-0.89)	0.79	1.50	0.005
p-value [a]	<.001			<.001			<.001			
Education										
<High school (HS)	0.55 (0.39-0.78)	0.53	‡	0.38 (0.25-0.59)	0.34	1.04	0.60 (0.48-0.75)	0.64	1.77	0.078
HS graduate/GED	0.64 (0.53-0.79)	0.56	‡	0.29 (0.24-0.36)	0.31	0.56	0.47 (0.36-0.59)	0.51	1.17	<.001
Some college/AA	0.52 (0.41-0.68)	0.51	2.67	0.34 (0.26-0.45)	0.33	1.42	0.39 (0.34-0.44)	0.41	1.38	0.001
≥College graduate	0.40 (0.37-0.44)	0.41	1.02	0.29 (0.26-0.33)	0.27	0.88	0.41 (0.35-0.47)	0.40	1.33	<.001
p-value [a]	<.001			0.521			0.003			
Household Income										
<$20,000	0.65 (0.45-0.94)	0.56	‡	0.30 (0.21-0.43)	0.30	‡	0.61 (0.50-0.76)	0.60	1.58	0.006
$20,000-<$50,000	0.60 (0.55-0.65)	0.55	1.57	0.32 (0.26-0.39)	0.31	0.74	0.45 (0.38-0.53)	0.46	1.44	<.001
$50,000-<$75,000	0.40 (0.24-0.67)	0.48	‡	0.35 (0.25-0.50)	0.32	1.16	0.44 (0.33-0.58)	0.43	1.28	0.572
≥$75,000	0.35 (0.32-0.38)	0.35	0.86	0.30 (0.27-0.34)	0.29	0.97	0.37 (0.33-0.42)	0.36	1.12	0.012
p-value [a]	<.001			0.864			0.001			
Poverty to Income Ratio										
≤ Median (1.63)	0.64 (0.47-0.88)	0.56	2.68	0.34 (0.25-0.45)	0.31	0.74	0.49 (0.42-0.58)	0.51	1.65	0.014
> Median (1.63)	0.40 (0.36-0.45)	0.41	1.13	0.31 (0.28-0.34)	0.30	1.02	0.40 (0.36-0.45)	0.40	1.27	<.001
	0.019			0.540			0.006			
Birthplace										
U.S.	0.27 (0.20-0.37)	0.27	0.75	0.17 (0.15-0.19)	0.16	0.30	0.25 (0.21-0.31)	0.25	0.80	0.002
Outside U.S.	0.55 (0.50-0.60)	0.55	1.48	0.35 (0.32-0.39)	0.32	1.03	0.53 (0.48-0.59)	0.54	1.52	<.001
p-value [a]	<.001			<.001			<.001			
BMI										
Underweight	0.57 (0.37-0.88)	0.46	‡	0.21 (0.13-0.33)	0.16	‡	0.47 (0.33-0.69)	0.52	1.38	0.003
Normal	0.45 (0.40-0.51)	0.44	1.40	0.29 (0.26-0.34)	0.29	0.88	0.43 (0.37-0.50)	0.42	1.49	<.001
Overweight	0.44 (0.33-0.60)	0.47	1.45	0.34 (0.30-0.38)	0.31	1.08	0.47 (0.39-0.56)	0.48	1.31	0.006
Obese	0.41 (0.33-0.50)	0.35	‡	0.34 (0.27-0.44)	0.28	1.41	0.37 (0.29-0.46)	0.31	1.86	0.604
p-value [a]	0.431			0.098			0.332			
Smoking (cotinine level)										
1st tertile	0.35 (0.30-0.41)	0.34	0.81	0.24 (0.21-0.26)	0.23	0.65	0.36 (0.32-0.41)	0.35	1.11	<.001
2nd tertile	0.46 (0.41-0.52)	0.50	1.16	0.31 (0.27-0.35)	0.31	0.85	0.45 (0.39-0.52)	0.47	1.28	<.001
3rd tertile	0.76 (0.54-1.08)	0.55	3.90	0.56 (0.43-0.74)	0.53	1.45	0.60 (0.49-0.74)	0.63	2.20	0.314
p-value [a]	<.001			<.001			<.001			
Fish Consumption (eaten during past 30 days)										
Yes	0.43 (0.37-0.49)	0.44	1.28	0.28 (0.24-0.32)	0.26	0.98	0.44 (0.39-0.49)	0.45	1.40	<.001
No	0.35 (0.24-0.49)	0.33	‡	0.33 (0.29-0.38)	0.31	0.99	0.28 (0.22-0.36)	0.29	1.12	0.524
p-value [a]	0.274			0.092			<.001			

† Not calculated due to high frequency of non-detected results (below limit of detection). ‡ Not calculated due to small number of samples. Raw Sample Counts are not provided for the restricted data. [a] Significance of difference in geometric mean across categories within covariate. [b] Significance of difference in geometric mean across Asian subgroups.

Table S7. Weighted statistics of blood lead levels (µg/dL) by Asian subgroup

	Chinese			Asian Indian			Other Asian			p-value[b]
	GM (95% CI)	Percentile 50th	Percentile 95th	GM (95% CI)	Percentile 50th	Percentile 95th	GM (95% CI)	Percentile 50th	Percentile 95th	
Overall	1.22 (1.08-1.38)	1.26	2.76	1.29 (1.15-1.44)	1.28	4.18	1.10 (0.97-1.23)	1.05	2.81	0.112
Sex										
Male	1.41 (1.21-1.65)	1.51	3.04	1.45 (1.27-1.65)	1.41	5.13	1.22 (1.05-1.41)	1.22	3.06	0.203
Female	1.06 (0.95-1.18)	1.11	2.46	1.15 (1.01-1.30)	1.13	3.87	1.00 (0.89-1.13)	0.98	2.72	0.110
p-value[a]	<.001			0.002			0.003			
Age										
6-11 yrs.	0.74 (0.63-0.87)	0.72	‡	0.64 (0.48-0.85)	0.61	‡	0.91 (0.68-1.22)	0.79	3.00	0.160
12-19 yrs.	0.63 (0.51-0.77)	0.57	1.15	0.90 (0.81-1.01)	0.81	1.93	0.69 (0.56-0.85)	0.68	1.75	<.001
20-39 yrs.	1.23 (0.95-1.60)	1.28	2.84	1.06 (0.87-1.29)	0.94	3.63	0.92 (0.81-1.05)	0.92	2.28	0.161
40-59 yrs.	1.49 (1.30-1.70)	1.49	3.30	1.65 (1.35-2.00)	1.47	4.27	1.39 (1.22-1.59)	1.37	3.08	0.328
60+ yrs.	1.52 (1.34-1.74)	1.50	2.51	2.19 (1.83-2.62)	1.75	5.95	1.53 (1.37-1.70)	1.52	3.22	0.003
p-value[a]	<.001			<.001			<.001			
Education										
<High school (HS)	1.39 (0.90-2.14)	1.47	‡	1.90 (1.50-2.41)	1.52	4.55	1.46 (1.28-1.67)	1.50	3.37	0.108
HS graduate/GED	1.42 (1.12-1.80)	1.39	‡	1.10 (0.75-1.60)	1.17	*	1.29 (1.05-1.57)	1.22	2.94	0.455
Some college/AA	1.45 (1.11-1.88)	1.41	3.29	1.04 (0.82-1.32)	0.89	2.28	1.02 (0.87-1.20)	0.98	2.47	0.038
≥College graduate	1.13 (1.01-1.26)	1.14	2.52	1.30 (1.15-1.47)	1.30	4.04	0.96 (0.85-1.09)	0.95	2.40	0.002
p-value[a]	0.065			0.032			<.001			
Household Income										
<$20,000	1.38 (1.02-1.88)	1.39	2.58	0.82 (0.52-1.29)	0.80	‡	1.20 (0.97-1.48)	1.26	3.14	0.153
$20,000-<$50,000	1.49 (1.18-1.89)	1.53	3.01	1.45 (1.20-1.74)	1.47	3.73	1.10 (0.96-1.25)	1.03	2.98	0.013
$50,000-<$75,000	1.19 (0.81-1.75)	1.39	‡	1.38 (1.12-1.72)	1.25	2.72	1.07 (0.85-1.34)	1.05	2.51	0.206
≥$75,000	1.06 (0.94-1.20)	0.97	2.49	1.33 (1.14-1.54)	1.23	4.94	1.04 (0.90-1.19)	1.00	2.73	0.022
p-value[a]	0.069			0.140			0.447			
Poverty to Income Ratio										
≤ Median (1.63)	1.48 (1.09-2.03)	1.46	2.81	1.06 (0.73-1.55)	1.14	3.20	1.19 (1.01-1.41)	1.17	3.25	0.380
> Median (1.63)	1.15 (1.02-1.29)	1.16	2.56	1.37 (1.26-1.48)	1.29	4.24	1.03 (0.91-1.17)	0.98	2.65	<.001
	0.125			0.185			0.123			
Birthplace										
U.S.	0.82 (0.72-0.93)	0.80	1.85	0.71 (0.53-0.94)	0.69	2.28	0.79 (0.72-0.88)	0.77	2.24	0.637
Outside U.S.	1.42 (1.29-1.57)	1.44	2.92	1.44 (1.32-1.58)	1.39	4.34	1.24 (1.10-1.40)	1.22	2.99	0.151
p-value[a]	<.001			<.001			<.001			
BMI										
Underweight	1.33 (0.88-2.00)	1.09	‡	0.65 (0.37-1.14)	0.84	‡	1.20 (0.96-1.50)	1.19	2.17	0.092
Normal	1.14 (1.02-1.28)	1.13	2.99	1.29 (1.13-1.48)	1.23	3.87	1.09 (0.96-1.24)	1.05	2.93	0.112
Overweight	1.45 (1.25-1.69)	1.51	2.60	1.41 (1.14-1.76)	1.28	4.56	1.15 (1.00-1.33)	1.18	2.74	0.085
Obese	1.58 (1.30-1.93)	1.51	‡	1.26 (0.93-1.72)	1.48	3.08	0.99 (0.80-1.22)	0.97	2.23	0.009
p-value[a]	<.001			0.065			0.358			
Smoking (cotinine level)										
1st tertile	1.02 (0.89-1.18)	0.95	2.47	0.87 (0.64-1.18)	0.81	4.12	0.94 (0.84-1.06)	0.93	2.18	0.541
2nd tertile	1.25 (1.11-1.41)	1.27	2.52	1.46 (1.31-1.63)	1.39	3.84	1.22 (1.09-1.38)	1.21	3.28	0.099
3rd tertile	1.77 (1.30-2.41)	1.79	3.04	1.69 (1.11-2.58)	1.87	5.36	1.24 (1.02-1.51)	1.14	3.20	0.180
p-value[a]	0.021			0.011			0.004			
Fish Consumption (eaten during past 30 days)										
Yes	1.20 (1.07-1.33)	1.17	2.82	1.22 (1.01-1.47)	1.22	4.26	1.11 (0.99-1.25)	1.06	2.73	0.427
No	1.01 (0.73-1.39)	1.14	‡	1.31 (1.17-1.47)	1.29	3.90	0.83 (0.67-1.02)	0.81	2.75	<.001
p-value[a]	0.221			0.496			<.001			

‡ Not calculated due to small number of samples. [a] Significance of difference in geometric mean across categories within covariate. [b] Significance of difference in geometric mean across Asian subgroups.

Table S8. Weighted statistics of blood mercury (total) levels (μg/L) by Asian subgroup

	Chinese			Asian Indian			Other Asian			p-value[b]
	GM (95% CI)	Percentile 50th	95th	GM (95% CI)	Percentile 50th	95th	GM (95% CI)	Percentile 50th	95th	
Overall	2.58 (2.08-3.20)	2.62	8.73	0.79 (0.55-1.13)	0.61	7.49	2.48 (2.08-2.95)	2.72	12.02	<.001
Sex										
Male	2.70 (2.13-3.44)	2.71	8.11	0.86 (0.58-1.27)	0.66	7.58	2.48 (2.00-3.07)	2.90	11.59	<.001
Female	2.47 (1.94-3.13)	2.54	10.18	0.73 (0.49-1.08)	0.49	6.17	2.48 (2.07-2.97)	2.65	12.26	<.001
p-value [a]	0.387			0.279			0.995			
Age										
6-11 yrs.	1.05 (0.80-1.37)	0.88	3.53	0.78 (0.46-1.34)	0.56	4.02	0.72 (0.53-0.98)	0.76	2.26	0.243
12-19 yrs.	1.42 (1.20-1.68)	1.52	4.60	0.78 (0.48-1.27)	0.68	4.86	1.11 (0.82-1.52)	1.21	5.02	0.052
20-39 yrs.	2.23 (1.56-3.18)	2.38	6.62	0.60 (0.40-0.92)	0.45	4.46	2.18 (1.88-2.53)	2.56	8.36	<.001
40-59 yrs.	3.98 (2.80-5.65)	4.23	9.43	1.18 (0.74-1.89)	1.10	12.31	3.93 (3.15-4.90)	3.88	18.12	<.001
60+ yrs.	3.52 (2.54-4.88)	3.77	10.69	0.62 (0.26-1.45)	0.42	5.35	3.80 (3.06-4.72)	3.84	12.32	<.001
p-value [a]	<.001			0.066			<.001			
Education										
<High school (HS)	2.84 (1.41-5.72)	3.04	‡	0.64 (0.26-1.56)	0.35	5.20	2.80 (2.00-3.94)	3.48	12.20	0.013
HS graduate/GED	2.80 (1.58-4.95)	2.52	‡	3.40 (2.30-5.04)	3.24	13.04	3.08 (2.10-4.52)	3.36	12.63	0.543
Some college/AA	2.89 (2.19-3.83)	3.37	8.03	0.74 (0.38-1.47)	0.88	4.20	2.33 (1.90-2.88)	2.59	10.23	<.001
≥College graduate	2.46 (1.93-3.12)	2.45	8.23	0.67 (0.44-1.01)	0.47	7.19	2.32 (1.83-2.92)	2.58	11.63	<.001
p-value [a]	0.810			<.001			0.404			
Household Income										
<$20,000	2.74 (1.57-4.78)	2.69	10.40	1.06 (0.47-2.41)	1.13	6.15	3.35 (2.33-4.82)	3.69	24.26	0.023
$20,000-<$50,000	2.45 (1.68-3.58)	2.49	10.14	0.58 (0.29-1.16)	0.39	3.43	2.18 (1.53-3.09)	2.50	10.44	<.001
$50,000-<$75,000	1.77 (1.23-2.54)	1.81	‡	0.85 (0.44-1.64)	0.57	6.68	2.22 (1.55-3.17)	2.58	7.14	0.036
≥$75,000	2.72 (2.19-3.39)	2.78	8.26	0.63 (0.40-0.98)	0.48	4.78	2.64 (2.10-3.33)	2.69	11.97	<.001
p-value [a]	0.124			0.251			0.258			
Poverty to Income Ratio										
≤ Median (1.63)	2.87 (1.85-4.47)	2.82	10.87	0.86 (0.34-2.14)	0.79	5.48	2.81 (2.10-3.76)	3.11	15.04	0.034
> Median (1.63)	2.50 (2.05-3.04)	2.49	8.28	0.65 (0.44-0.95)	0.49	4.90	2.36 (1.93-2.89)	2.62	11.21	<.001
	0.512			0.531			0.341			
Birthplace										
U.S.	2.25 (1.80-2.83)	2.27	6.55	0.88 (0.67-1.16)	0.87	4.46	1.51 (1.26-1.82)	1.66	7.01	<.001
Outside U.S.	2.72 (1.98-3.74)	2.64	8.82	0.77 (0.52-1.15)	0.50	7.82	2.99 (2.53-3.52)	3.28	13.70	<.001
p-value [a]	0.382			0.490			<.001			
BMI										
Underweight	2.72 (1.51-4.88)	2.88	‡	0.40 (0.21-0.76)	0.28	‡	1.75 (0.93-3.27)	2.61	5.23	<.001
Normal	2.38 (1.89-2.99)	2.38	8.45	0.79 (0.55-1.15)	0.60	6.46	2.56 (2.14-3.07)	2.93	12.01	<.001
Overweight	3.31 (2.45-4.49)	3.90	7.90	0.89 (0.51-1.56)	0.66	8.48	2.86 (2.25-3.62)	2.90	13.21	<.001
Obese	3.75 (2.22-6.36)	3.46	‡	0.76 (0.48-1.20)	0.81	3.22	1.73 (1.20-2.49)	1.77	6.35	<.001
p-value [a]	0.005			0.151			0.038			
Smoking (cotinine level)										
1st tertile	2.34 (1.87-2.92)	2.23	7.74	0.99 (0.69-1.43)	1.06	7.17	2.42 (2.08-2.82)	2.59	11.89	<.001
2nd tertile	2.77 (2.00-3.85)	2.85	9.60	0.65 (0.40-1.04)	0.43	5.85	2.61 (2.04-3.34)	3.09	11.33	<.001
3rd tertile	2.66 (1.70-4.15)	2.65	7.88	1.10 (0.74-1.64)	0.91	9.19	2.32 (1.69-3.17)	2.71	11.21	0.002
p-value [a]	0.684			0.125			0.620			
Fish Consumption (eaten during past 30 days)										
Yes	2.71 (2.20-3.34)	2.63	8.28	1.71 (1.38-2.10)	1.77	8.53	2.88 (2.46-3.37)	2.91	13.08	<.001
No	1.17 (0.77-1.76)	1.06	‡	0.30 (0.25-0.36)	0.27	1.12	0.74 (0.56-0.98)	0.83	4.37	<.001
p-value [a]	0.003			<.001			<.001			

‡ Not calculated due to small number of samples. [a] Significance of difference in geometric mean across categories within covariate. [b] Significance of difference in geometric mean across Asian subgroups.

Table S9. Weighted statistics of urinary arsenic (total) levels (μg/g-creatinine) by Asian subgroup

	Chinese			Asian Indian			Other Asian			p-value[b]
	GM (95% CI)	Percentile 50th	Percentile 95th	GM (95% CI)	Percentile 50th	Percentile 95th	GM (95% CI)	Percentile 50th	Percentile 95th	
Overall	23.1 (18.3-29.2)	20.3	111.0	10.9 (8.6-14.0)	9.9	35.0	28.2 (23.2-34.2)	26.7	167.7	<.001
Sex										
Male	26.3 (18.7-37.2)	20.5	136.0	8.8 (7.0-11.1)	8.0	24.9	23.8 (17.9-31.6)	22.6	162.9	<.001
Female	19.2 (15.4-24.0)	18.9	‡	14.0 (9.8-19.9)	11.4	49.2	31.9 (25.7-39.7)	31.2	171.1	<.001
p-value[a]	0.108			0.011			0.070			
Age										
6-11 yrs.	32.7 (17.3-61.8)	28.2	‡	13.6 (6.2-29.9)	11.9	‡	17.9 (11.6-27.5)	12.7	‡	0.152
12-19 yrs.	11.2 (6.5-19.1)	8.5	‡	6.8 (5.6-8.3)	6.1	‡	11.3 (8.1-15.9)	10.7	39.2	0.037
20-39 yrs.	20.8 (14.8-29.3)	19.7	57.2	11.3 (8.6-14.9)	10.1	35.4	24.2 (18.4-31.9)	19.7	148.7	0.003
40-59 yrs.	30.1 (21.0-43.2)	20.9	‡	12.9 (8.8-19.1)	11.0	48.1	37.5 (25.4-55.4)	34.8	172.8	<.001
60+ yrs.	24.0 (14.0-41.2)	16.2	‡	8.0 (4.4-14.6)	7.9	‡	52.8 (42.8-65.1)	41.2	166.6	<.001
p-value[a]	0.025			0.037			<.001			
Education										
<High school (HS)	18.5 (9.2-37.4)	14.4	‡	10.1 (4.9-21.1)	6.9	‡	38.3 (23.7-62.0)	38.5	163.8	0.007
HS graduate/GED	32.7 (17.4-61.2)	24.2	‡	16.6 (10.2-26.9)	19.9	‡	21.2 (15.7-28.7)	19.9	105.0	0.323
Some college/AA	25.3 (14.8-43.3)	23.6	61.1	8.6 (3.7-19.8)	5.6	‡	28.5 (21.8-37.2)	24.5	311.5	0.035
≥College graduate	20.5 (15.4-27.2)	13.8	94.0	10.9 (8.5-14.0)	11.3	26.5	27.1 (20.8-35.3)	25.8	175.3	<.001
p-value[a]	0.436			0.363			0.114			
Household Income										
<$20,000	39.8 (28.6-55.4)	32.3	‡	16.9 (8.3-34.5)	21.4	‡	38.5 (26.7-55.5)	35.1	166.4	0.027
$20,000-<$50,000	28.0 (22.2-35.3)	23.3	92.6	9.4 (6.2-14.4)	8.0	‡	23.3 (18.6-29.3)	22.9	126.2	<.001
≥$75,000	17.6 (14.1-22.0)	13.4	68.1	9.5 (7.6-11.9)	9.4	23.2	27.9 (19.5-39.9)	23.8	165.0	<.001
p-value[a]	<.001			0.251			0.114			
Poverty to Income Ratio										
≤ Median (1.63)	39.4 (31.4-49.5)	33.0	198.1	14.3 (6.5-31.5)	18.3	‡	30.3 (23.2-39.5)	31.8	138.0	0.055
> Median (1.63)	18.8 (15.8-22.2)	14.6	76.3	9.7 (7.9-11.9)	9.4	25.8	25.8 (21.0-31.8)	22.8	163.2	<.001
	<.001			0.313			0.358			
Birthplace										
U.S.	17.1 (10.1-29.1)	13.7	56.2	9.0 (6.4-12.7)	7.9	‡	16.5 (11.9-23.0)	13.7	174.2	0.009
Outside U.S.	25.2 (19.6-32.5)	21.8	113.0	11.4 (8.6-15.0)	10.1	39.2	33.7 (27.2-41.9)	33.3	166.9	<.001
p-value[a]	0.163			0.284			<.001			
BMI										
Underweight	‡	‡	‡	‡	‡	‡	‡	‡	‡	
Normal	21.0 (16.4-27.0)	16.5	74.9	10.8 (8.5-13.7)	9.7	29.4	31.0 (23.9-40.5)	30.6	167.6	<.001
Overweight	32.1 (18.5-55.7)	28.9	154.1	11.4 (8.2-15.7)	10.1	34.4	25.7 (20.9-31.6)	22.9	123.1	<.001
Obese	‡	‡	‡	‡	‡	‡	‡	‡	‡	
p-value[a]	0.001			<.001			0.013			
Smoking (cotinine level)										
1st tertile	23.7 (17.4-32.4)	25.7	68.3	13.1 (8.8-19.3)	10.1	‡	27.2 (19.1-38.6)	28.1	113.7	<.001
2nd tertile	20.8 (14.3-30.4)	17.1	112.4	10.2 (7.6-13.6)	9.4	40.0	27.9 (21.1-36.8)	27.2	185.4	<.001
3rd tertile	25.5 (13.6-47.9)	20.6	‡	8.9 (6.4-12.5)	5.6	‡	29.6 (19.7-44.3)	23.5	157.7	<.001
p-value[a]	0.705			0.346			0.953			
Fish Consumption (eaten during past 30 days)										
Yes	21.1 (17.0-26.2)	17.2	89.5	15.1 (11.2-20.2)	12.9	46.7	29.9 (23.8-37.6)	27.2	167.0	0.001
No	8.7 (5.2-14.6)	9.4	‡	8.0 (6.1-10.3)	6.7	29.0	10.2 (6.8-15.2)	7.8	39.4	0.558
p-value[a]	0.005			0.002			<.001			

‡ Not calculated due to small number of samples. [a] Significance of difference in geometric mean across categories within covariate. [b] Significance of difference in geometric mean across Asian subgroups.

Table S10. Weighted statistics of urinary dimethylarsonic acid (DMA) levels (µg/g-creatinine) by Asian subgroup

	Chinese			Asian Indian			Other Asian			p-value [b]
	GM (95% CI)	Percentile 50th	95th	GM (95% CI)	Percentile 50th	95th	GM (95% CI)	Percentile 50th	95th	
Overall	9.8 (8.2-11.8)	9.8	27.26	6.3 (5.2-7.5)	6.2	17.82	11.6 (9.8-13.7)	10.8	55.73	<.001
Sex										
Male	9.7 (7.5-12.6)	9.4	31.20	5.0 (4.1-6.1)	4.5	11.49	10.1 (8.1-12.5)	10.2	38.82	<.001
Female	10.0 (8.1-12.4)	10.1	‡	8.1 (6.4-10.3)	7.9	18.15	12.8 (10.3-16.0)	11.2	61.04	0.010
p-value [a]	0.825			<.001			0.081			
Age										
6-11 yrs.	13.6 (8.8-20.9)	13.8	‡	10.9 (5.3-22.4)	9.7	‡	10.8 (7.0-16.5)	8.3	‡	0.693
12-19 yrs.	6.2 (3.7-10.4)	4.6	‡	3.9 (3.1-4.8)	3.3	‡	6.4 (5.3-7.8)	6.2	17.35	0.006
20-39 yrs.	8.3 (6.1-11.4)	9.1	20.80	6.2 (4.9-7.9)	6.2	12.34	9.6 (7.9-11.5)	8.8	53.02	0.054
40-59 yrs.	11.0 (8.9-13.5)	9.8	‡	7.4 (5.3-10.2)	7.2	‡	14.0 (10.1-19.5)	12.2	56.89	0.006
60+ yrs.	12.8 (8.4-19.5)	9.8	‡	4.7 (3.7-6.0)	4.5	‡	18.4 (14.8-22.8)	17.2	55.36	<.001
p-value [a]	0.118			<.001			<.001			
Education										
<High school (HS)	9.2 (5.1-16.8)	10.4	‡	5.6 (3.7-8.4)	5.4	‡	17.6 (11.2-27.7)	18.4	75.13	0.001
HS graduate/GED	12.0 (8.1-17.7)	12.0	‡	8.3 (6.5-10.7)	10.0	‡	10.2 (8.1-12.9)	10.8	‡	0.311
Some college/AA	11.0 (7.6-16.0)	9.7	23.92	5.2 (2.2-12.1)	2.9	‡	10.8 (8.6-13.6)	9.6	57.32	0.226
≥College graduate	8.8 (6.8-11.4)	7.6	31.59	6.4 (5.3-7.8)	5.8	15.95	10.2 (8.5-12.3)	9.6	47.51	<.001
p-value [a]	0.495			0.257			0.131			
Household Income										
<$20,000	16.0 (10.5-24.4)	14.5	‡	8.3 (4.5-15.3)	8.2	‡	17.5 (11.6-26.6)	14.5	80.19	0.002
$20,000-<$50,000	10.6 (8.1-13.9)	11.5	20.90	5.9 (3.7-9.3)	5.2	‡	10.4 (8.4-13.0)	10.7	47.38	0.033
≥$75,000	8.2 (6.2-10.8)	8.2	29.34	5.8 (4.7-7.0)	5.4	12.73	10.9 (8.5-14.1)	9.5	50.95	0.001
p-value [a]	0.033			0.546			0.131			
Poverty to Income Ratio										
≤ Median (1.63)	13.8 (9.6-19.8)	13.4	30.23	7.0 (3.5-13.9)	7.5	‡	14.6 (11.0-19.3)	13.5	79.49	0.013
> Median (1.63)	8.6 (7.2-10.2)	9.3	25.19	5.9 (5.0-7.1)	5.4	14.44	10.4 (8.7-12.5)	10.1	48.94	<.001
	0.019			0.629			0.052			
Birthplace										
U.S.	8.2 (5.7-11.8)	8.4	18.03	5.1 (3.9-6.7)	3.9	‡	7.9 (6.3-9.8)	7.4	31.71	0.003
Outside U.S.	10.4 (8.3-13.1)	10.0	28.62	6.5 (5.3-8.1)	6.5	16.85	13.2 (10.9-15.9)	11.8	59.02	<.001
p-value [a]	0.257			0.138			<.001			
BMI										
Underweight	‡	‡	‡	‡	‡	‡	‡	‡	‡	
Normal	9.2 (7.6-11.1)	9.6	28.76	5.9 (4.9-7.2)	5.7	12.98	12.3 (9.9-15.2)	10.8	56.81	<.001
Overweight	12.8 (8.2-20.1)	12.3	25.04	6.7 (5.2-8.6)	7.7	17.85	10.7 (8.9-12.8)	11.0	34.49	0.014
Obese	‡	‡	‡	‡	‡	‡	‡	‡	‡	
p-value [a]	<.001			0.108			0.111			
Smoking (cotinine level)										
1st tertile	10.2 (7.6-13.6)	9.8	34.81	6.7 (4.7-9.6)	6.0	‡	11.8 (8.6-16.2)	10.6	49.73	<.001
2nd tertile	9.2 (6.7-12.5)	9.4	23.79	6.3 (5.0-7.9)	6.2	15.85	11.4 (8.7-14.9)	10.8	53.88	0.008
3rd tertile	11.0 (7.7-15.8)	9.6	‡	4.2 (3.3-5.4)	2.7	‡	10.1 (7.4-13.8)	9.7	56.07	<.001
p-value [a]	0.589			0.016			0.802			
Fish Consumption (eaten during past 30 days)										
Yes	9.2 (7.7-11.1)	9.4	30.14	7.4 (5.7-9.6)	7.2	‡	11.1 (9.4-13.2)	10.6	47.53	0.017
No	4.7 (2.9-7.4)	3.6	‡	5.2 (4.1-6.6)	4.8	12.89	6.5 (4.9-8.5)	5.8	22.79	0.475
p-value [a]	0.014			0.032			<.001			

‡ Not calculated due to small number of samples. [a] Significance of difference in geometric mean across categories within covariate. [b] Significance of difference in geometric mean across Asian subgroups.
